# Supplementary material for: Metformin modulates the unfolded protein responses, altering lifespan and health-promoting effects in UPR-activated worms
Source: PLoS One. 2025 Jun 16;20(6):e0326100. doi: 10.1371/journal.pone.0326100 (PMC12169583; doi:10.1371/journal.pone.0326100)
Supplement: S1 Fig — (PDF) [file pone.0326100.s001.pdf]

SUPPLEMENTARY DATA

Supplementary Figure S1

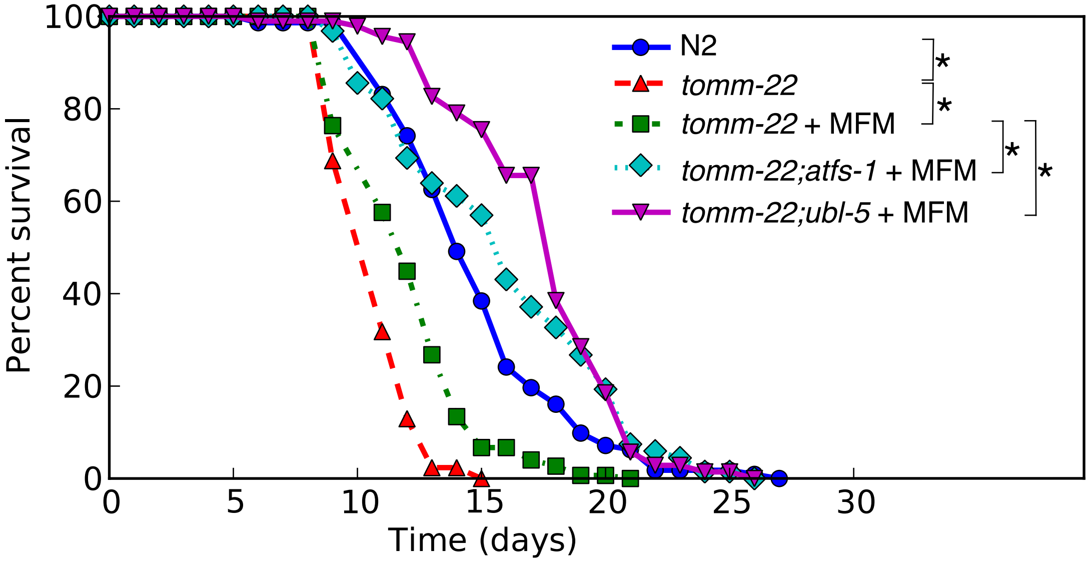

Supplementary Figure S1. Inactivation of UPR^{mt} by *atfs-1* and *ubl-1* contributes to longevity observed in metformin-treated *tomm-22*. Lifespan assays were conducted in wild-type N2 and *tomm-22* RNAi knockdown worms (UPR^{mt} activation). *tomm-22* nematodes were subjected to 50 mM metformin (MFM) treatment, and RNAi knockdown of *atfs-1* and *ubl-5* as indicated. Each assay consisted of 100-200 age-synchronized worms. Statistical analysis was performed using log-ranked test. * denotes statistical significance where Bonferroni's p-value < 0.01.
