# Supplementary figures and images for: Metformin modulates the unfolded protein responses, altering lifespan and health-promoting effects in UPR-activated worms
Source: PLoS One. 2025 Jun 16;20(6):e0326100. doi: 10.1371/journal.pone.0326100 (PMC12169583; doi:10.1371/journal.pone.0326100)

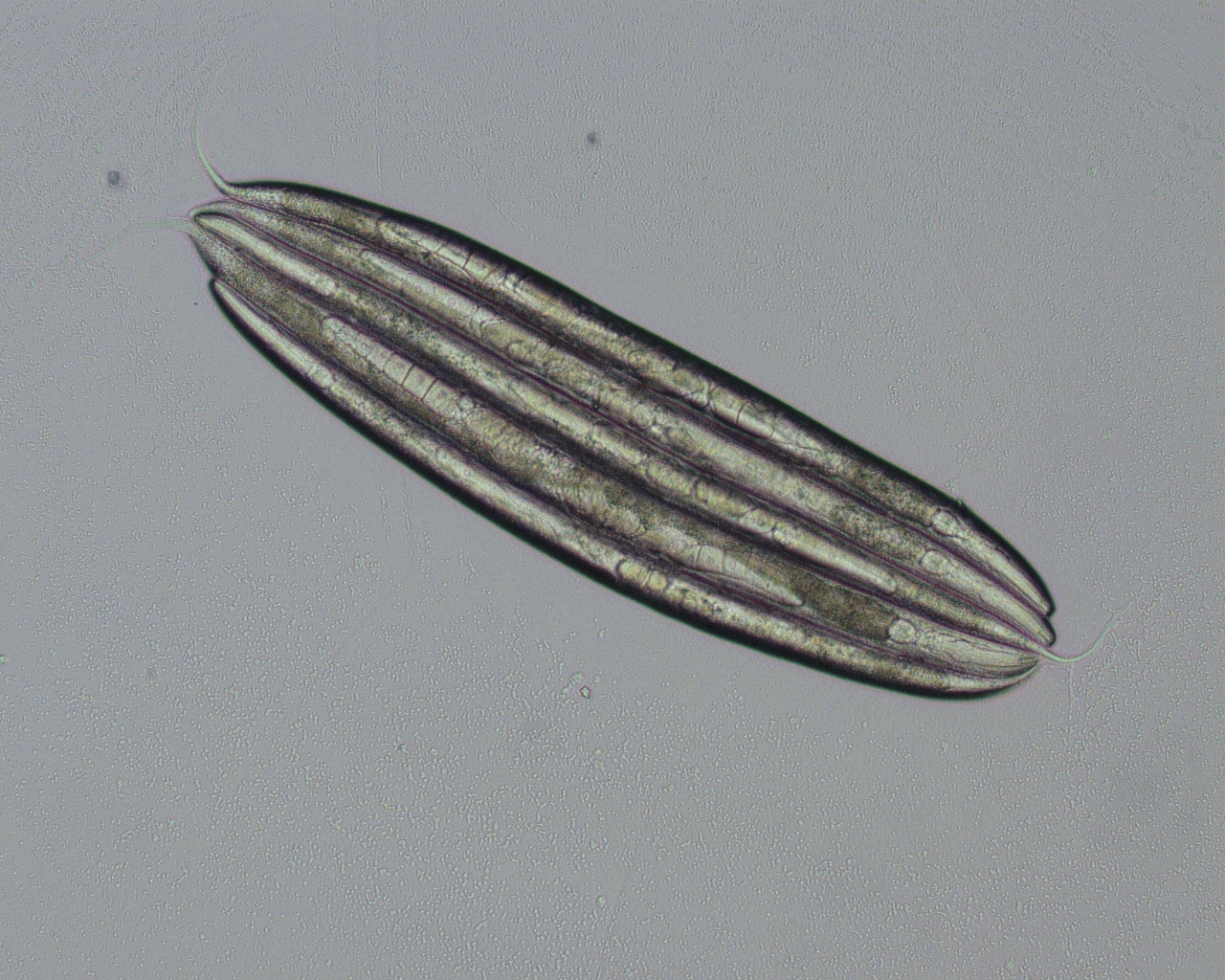

Supplement: S1 File — (ZIP) [file pone.0326100.s002.zip › Raw data of each experiment/Raw data Figure 2- Metformin effect on UPR/mdt-15/mdt-15 with MFM (BF).tif]

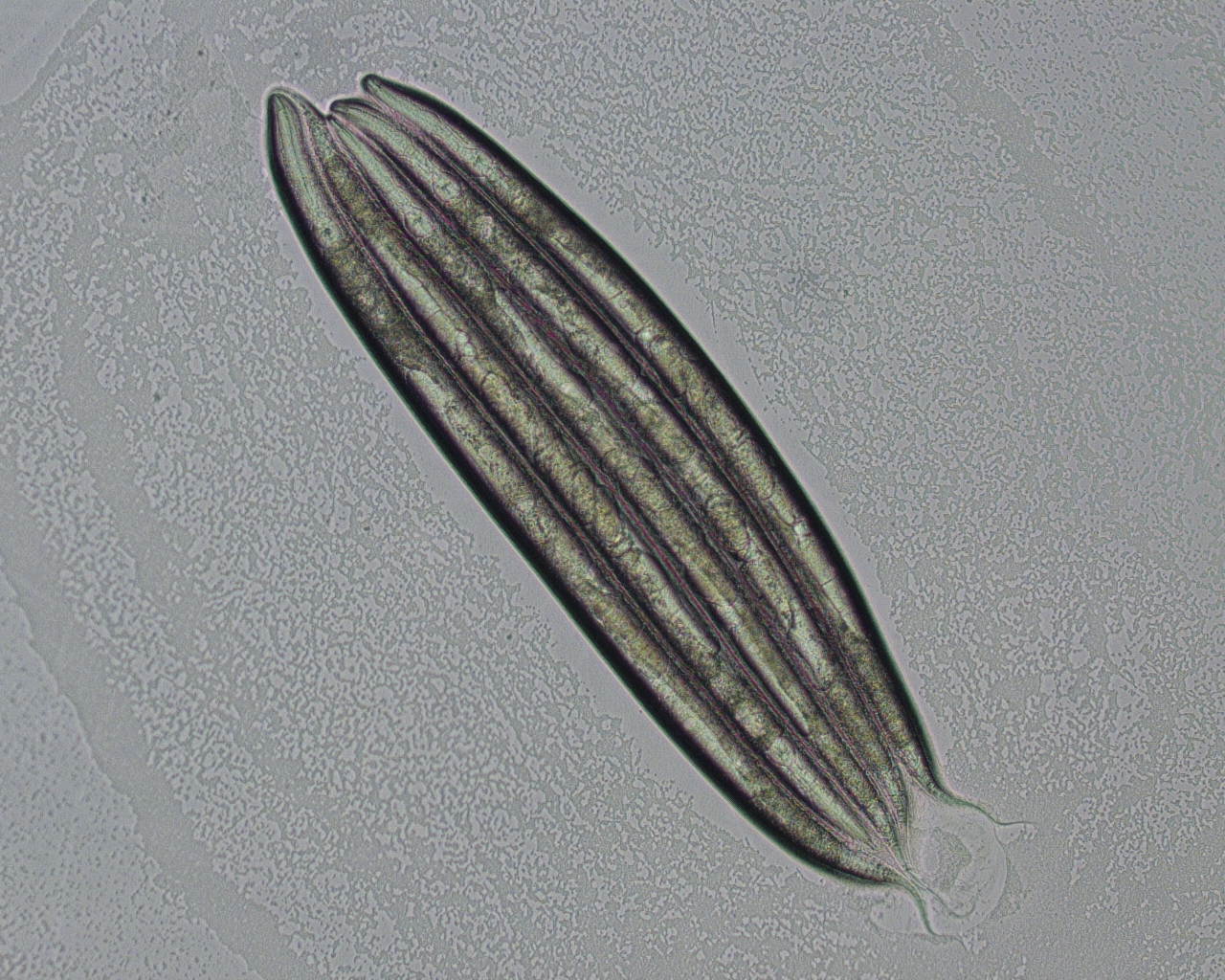

Supplement: S1 File — (ZIP) [file pone.0326100.s002.zip › Raw data of each experiment/Raw data Figure 2- Metformin effect on UPR/mdt-15/mdt-15 (BF).tif]

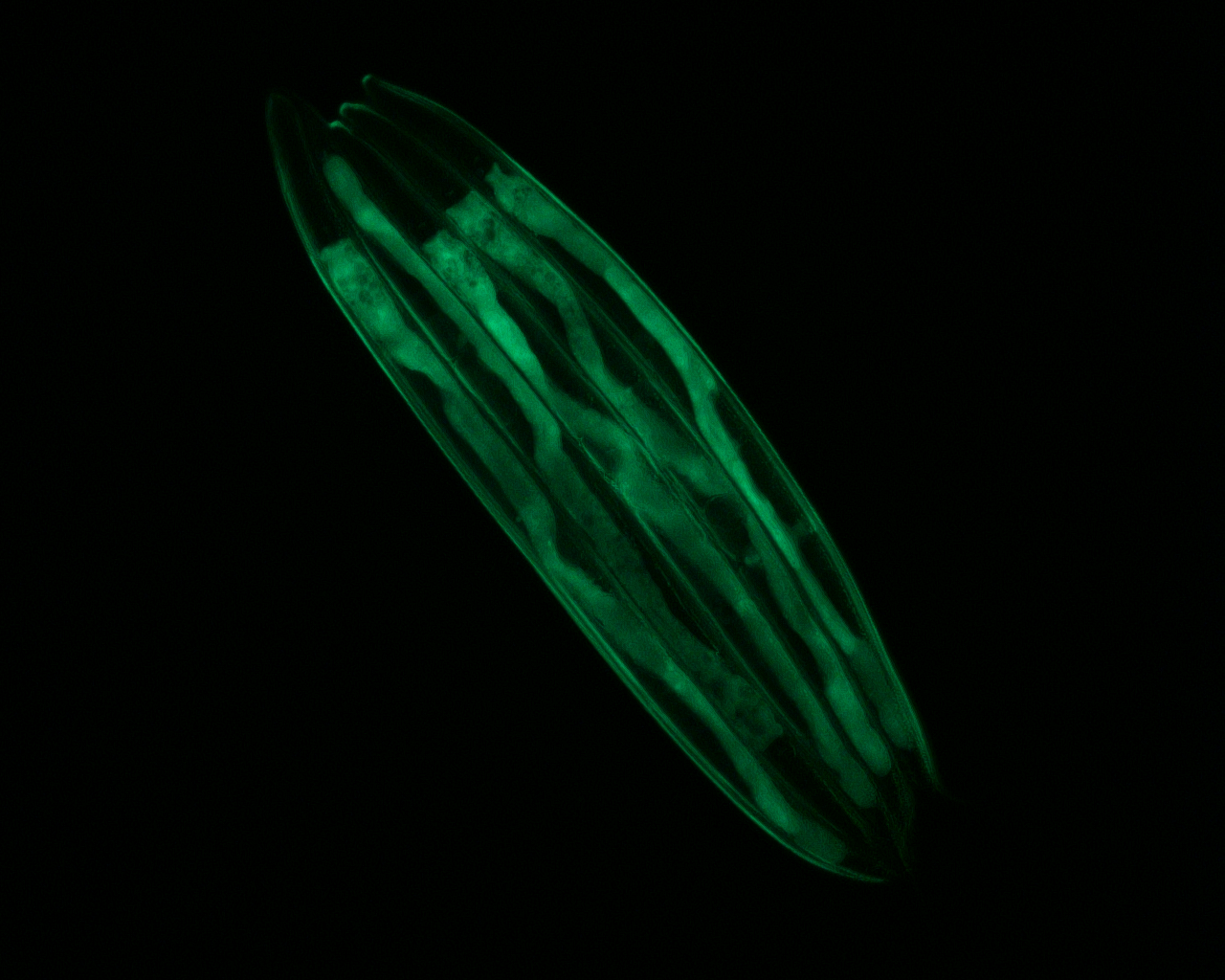

Supplement: S1 File — (ZIP) [file pone.0326100.s002.zip › Raw data of each experiment/Raw data Figure 2- Metformin effect on UPR/mdt-15/mdt-15 (GFP).tif]

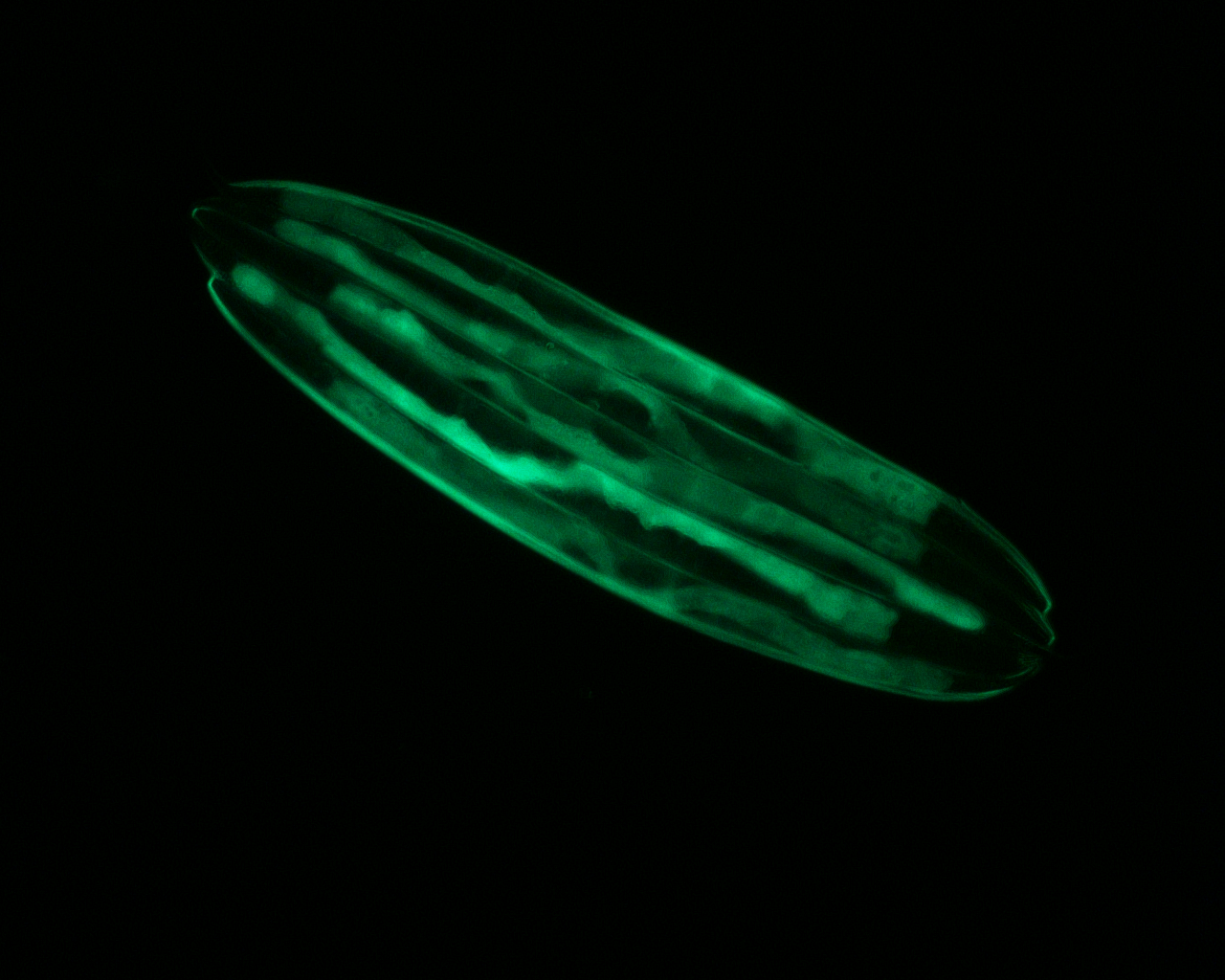

Supplement: S1 File — (ZIP) [file pone.0326100.s002.zip › Raw data of each experiment/Raw data Figure 2- Metformin effect on UPR/mdt-15/mdt-15 with MFM (GFP).tif]

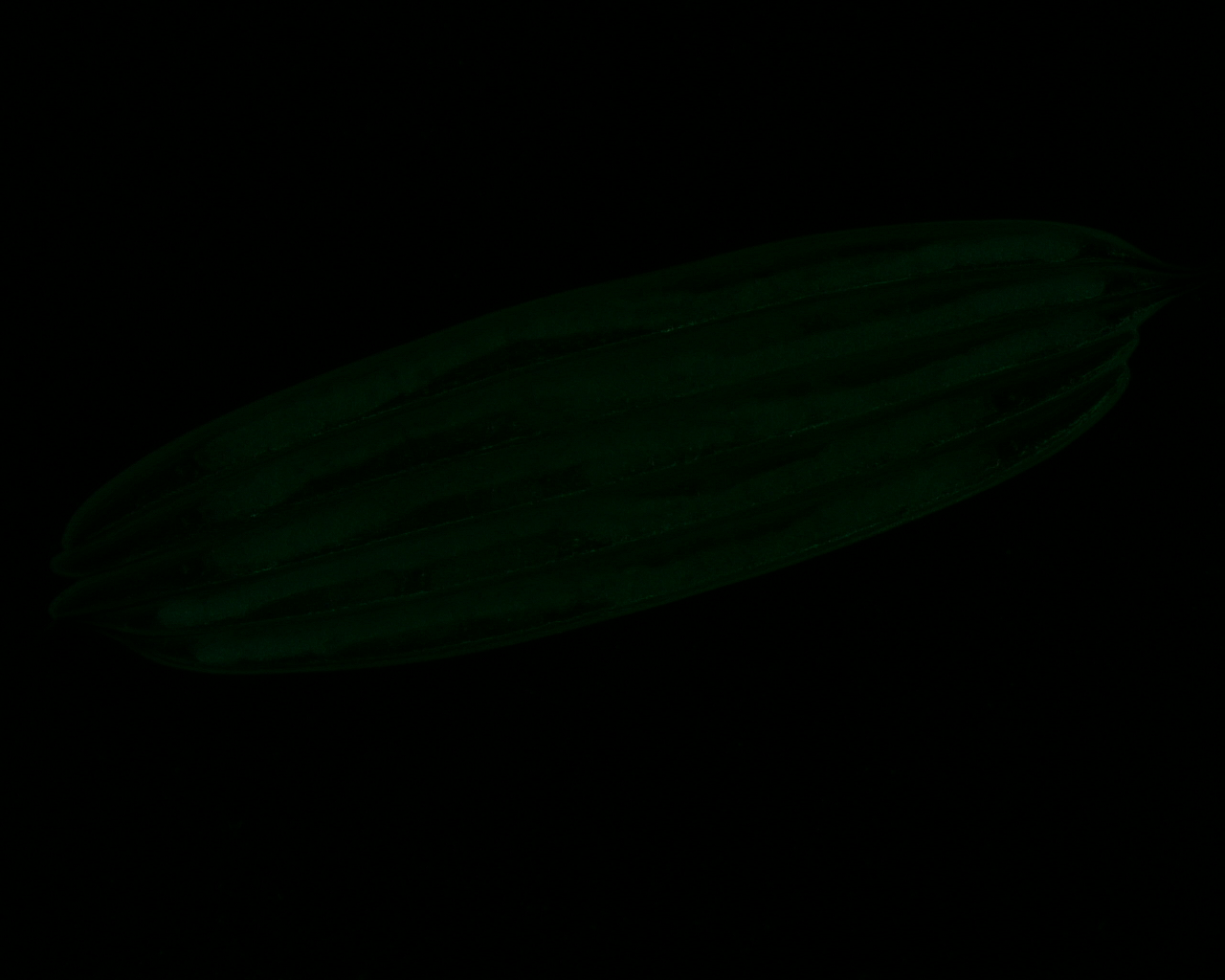

Supplement: S1 File — (ZIP) [file pone.0326100.s002.zip › Raw data of each experiment/Raw data Figure 2- Metformin effect on UPR/hsp-4 GFP (UPRer)/hsp-4 (GFP).tif]

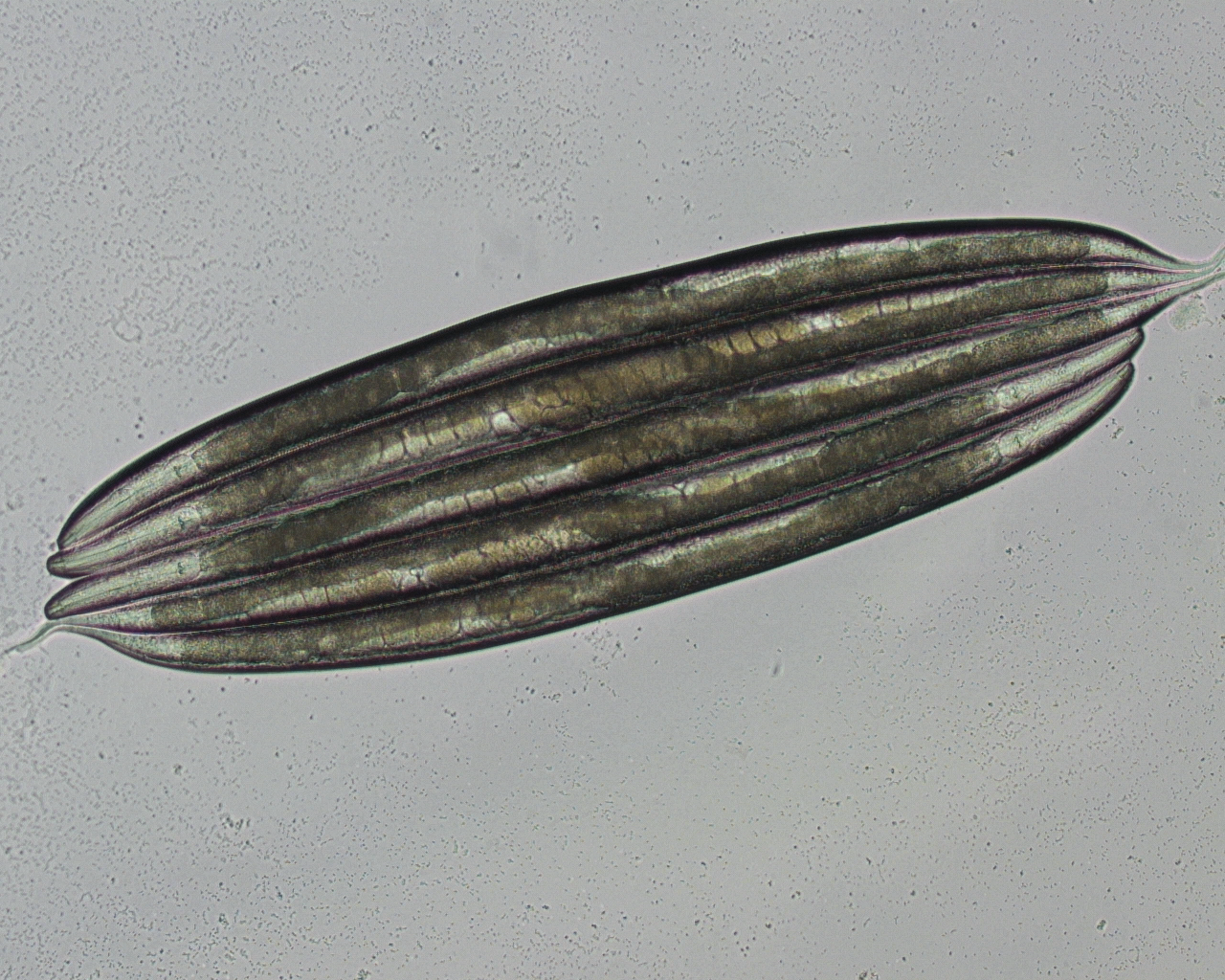

Supplement: S1 File — (ZIP) [file pone.0326100.s002.zip › Raw data of each experiment/Raw data Figure 2- Metformin effect on UPR/hsp-4 GFP (UPRer)/hsp-4 (BF).tif]

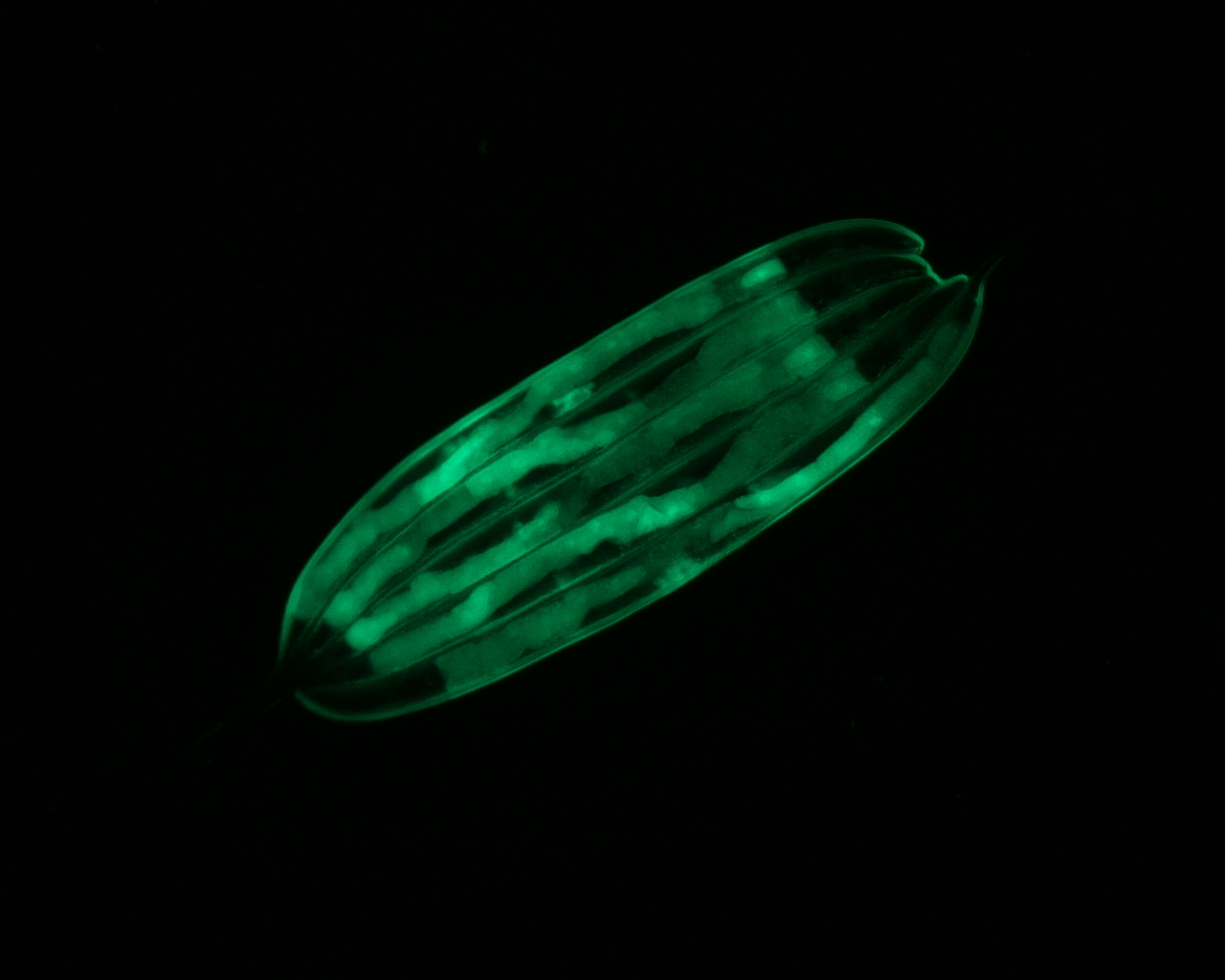

Supplement: S1 File — (ZIP) [file pone.0326100.s002.zip › Raw data of each experiment/Raw data Figure 2- Metformin effect on UPR/tag-335/tag-335 + MFM (GFP).tif]

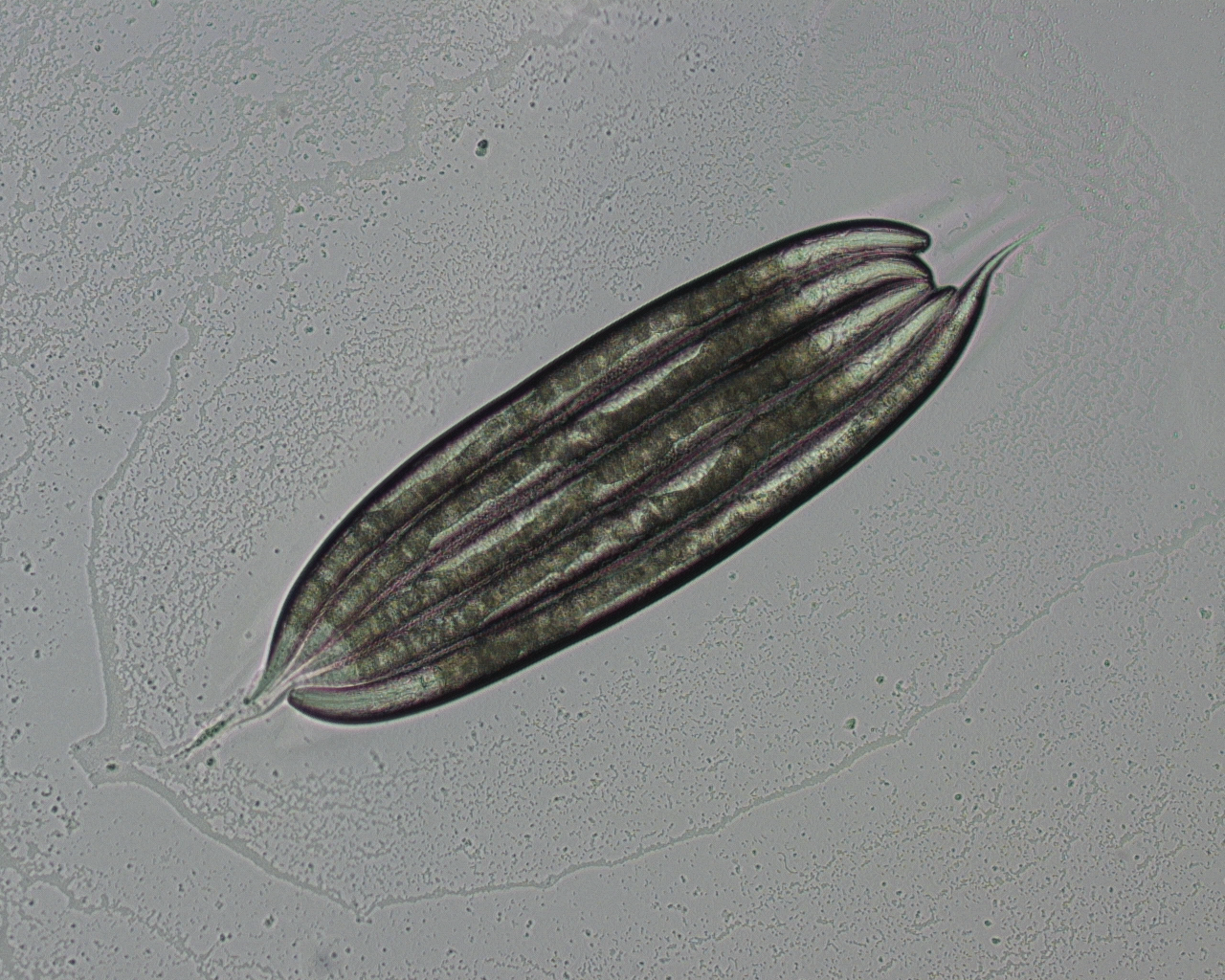

Supplement: S1 File — (ZIP) [file pone.0326100.s002.zip › Raw data of each experiment/Raw data Figure 2- Metformin effect on UPR/tag-335/tag-335 + MFM (BF).tif]

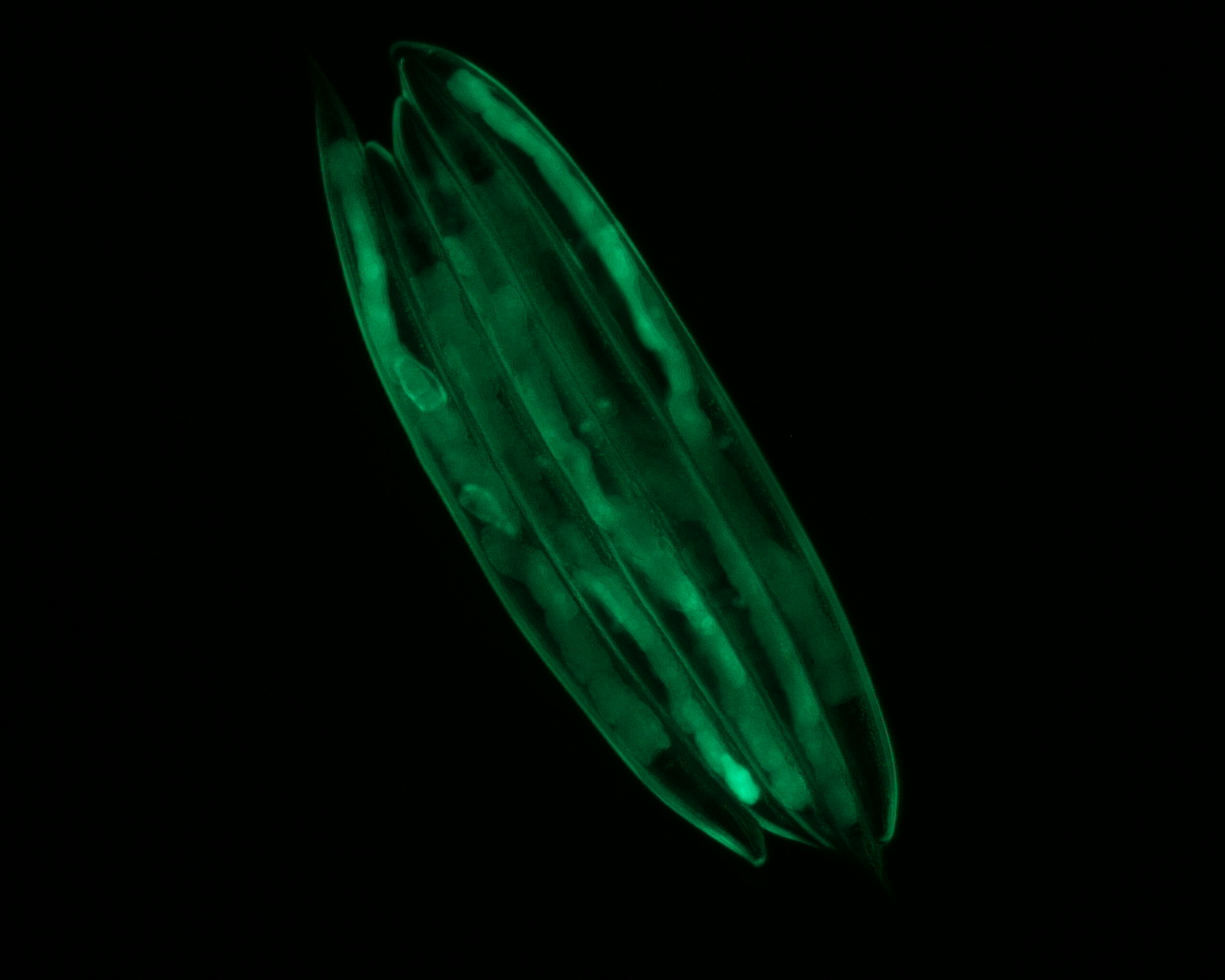

Supplement: S1 File — (ZIP) [file pone.0326100.s002.zip › Raw data of each experiment/Raw data Figure 2- Metformin effect on UPR/tag-335/tag-335 (GFP).tif]

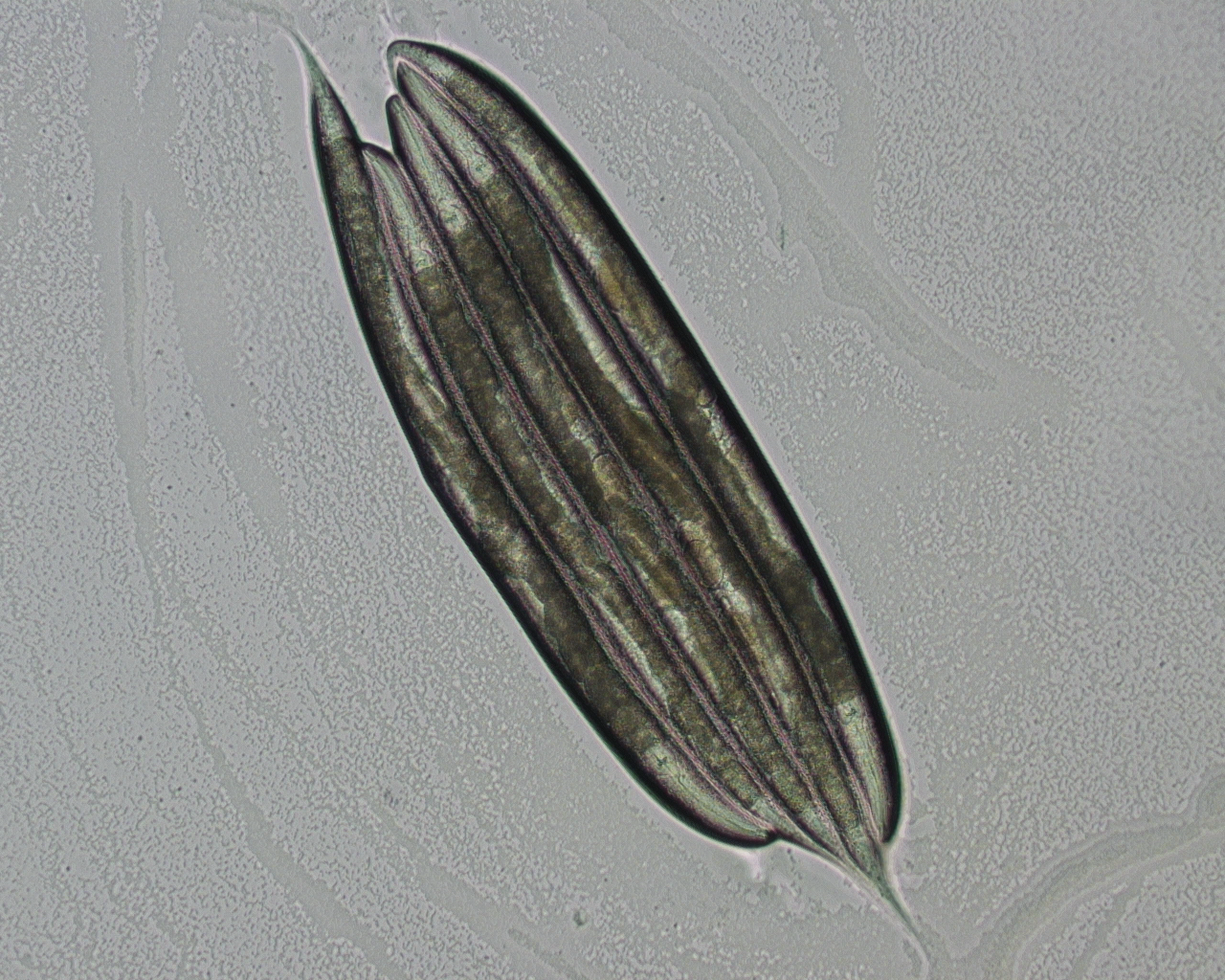

Supplement: S1 File — (ZIP) [file pone.0326100.s002.zip › Raw data of each experiment/Raw data Figure 2- Metformin effect on UPR/tag-335/tag-335 (BF).tif]

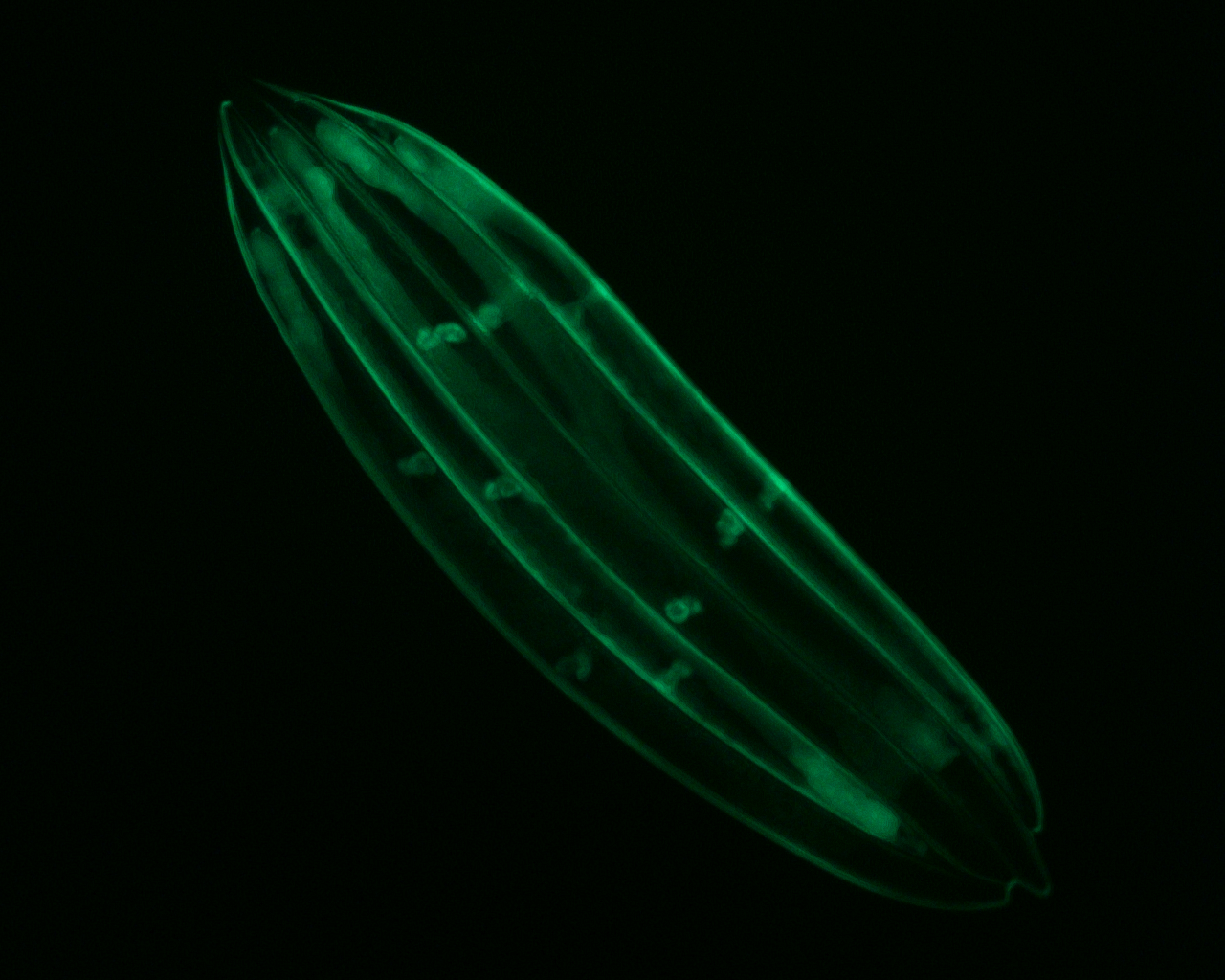

Supplement: S1 File — (ZIP) [file pone.0326100.s002.zip › Raw data of each experiment/Raw data Figure 2- Metformin effect on UPR/tmem-131/tmem-131 (GFP).tif]

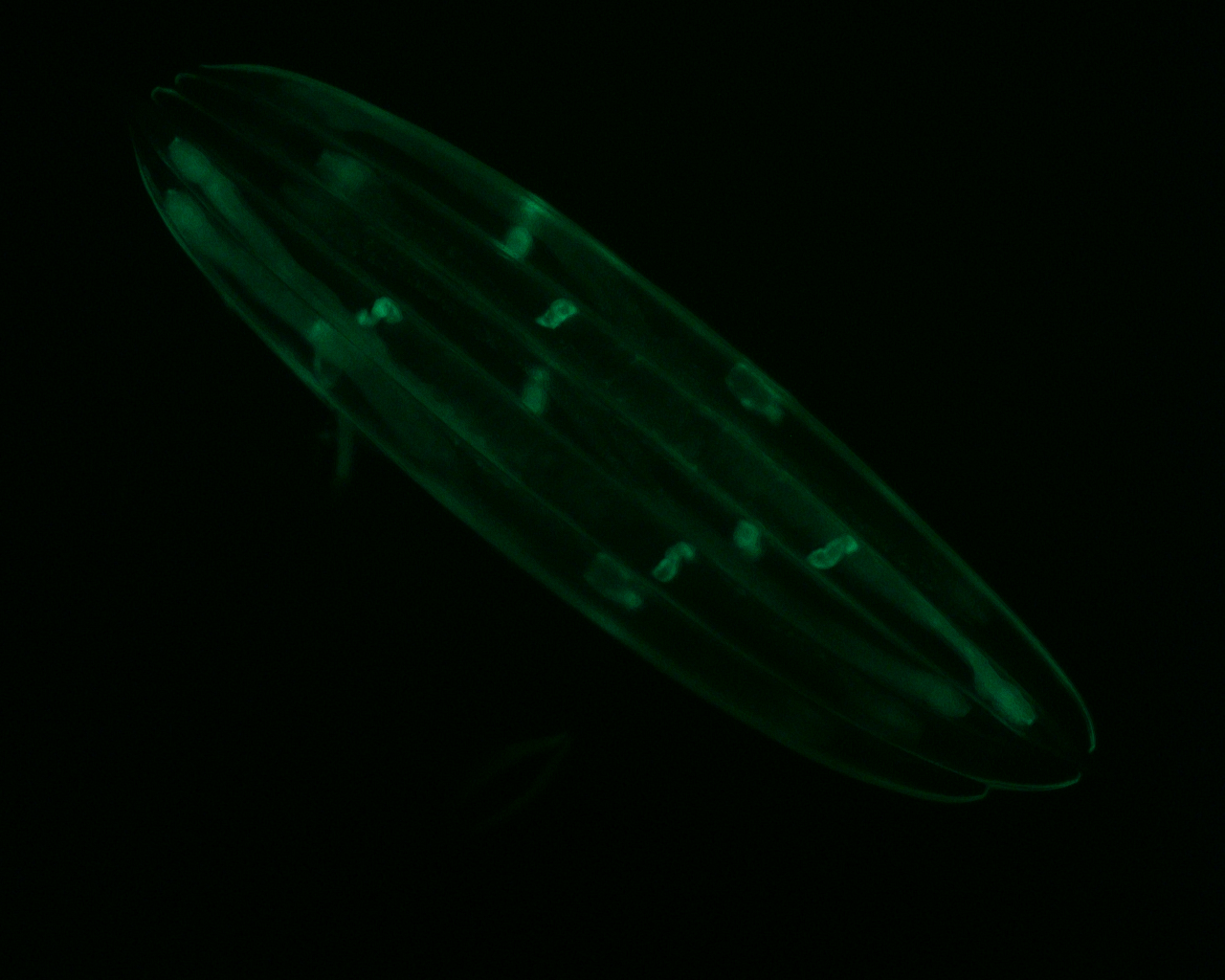

Supplement: S1 File — (ZIP) [file pone.0326100.s002.zip › Raw data of each experiment/Raw data Figure 2- Metformin effect on UPR/tmem-131/tmem-131 + MFM (GFP).tif]

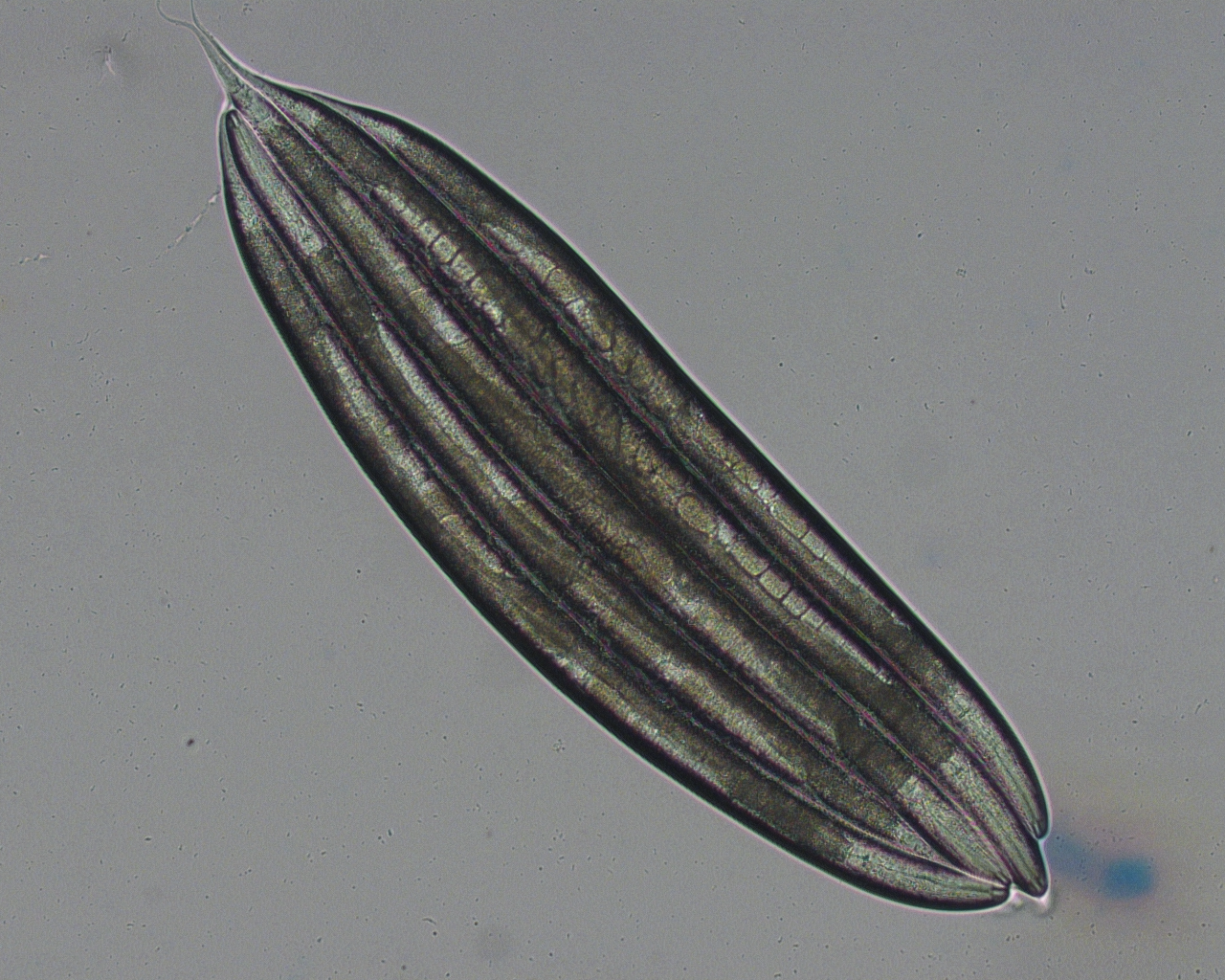

Supplement: S1 File — (ZIP) [file pone.0326100.s002.zip › Raw data of each experiment/Raw data Figure 2- Metformin effect on UPR/tmem-131/tmem-131 (BF).tif]

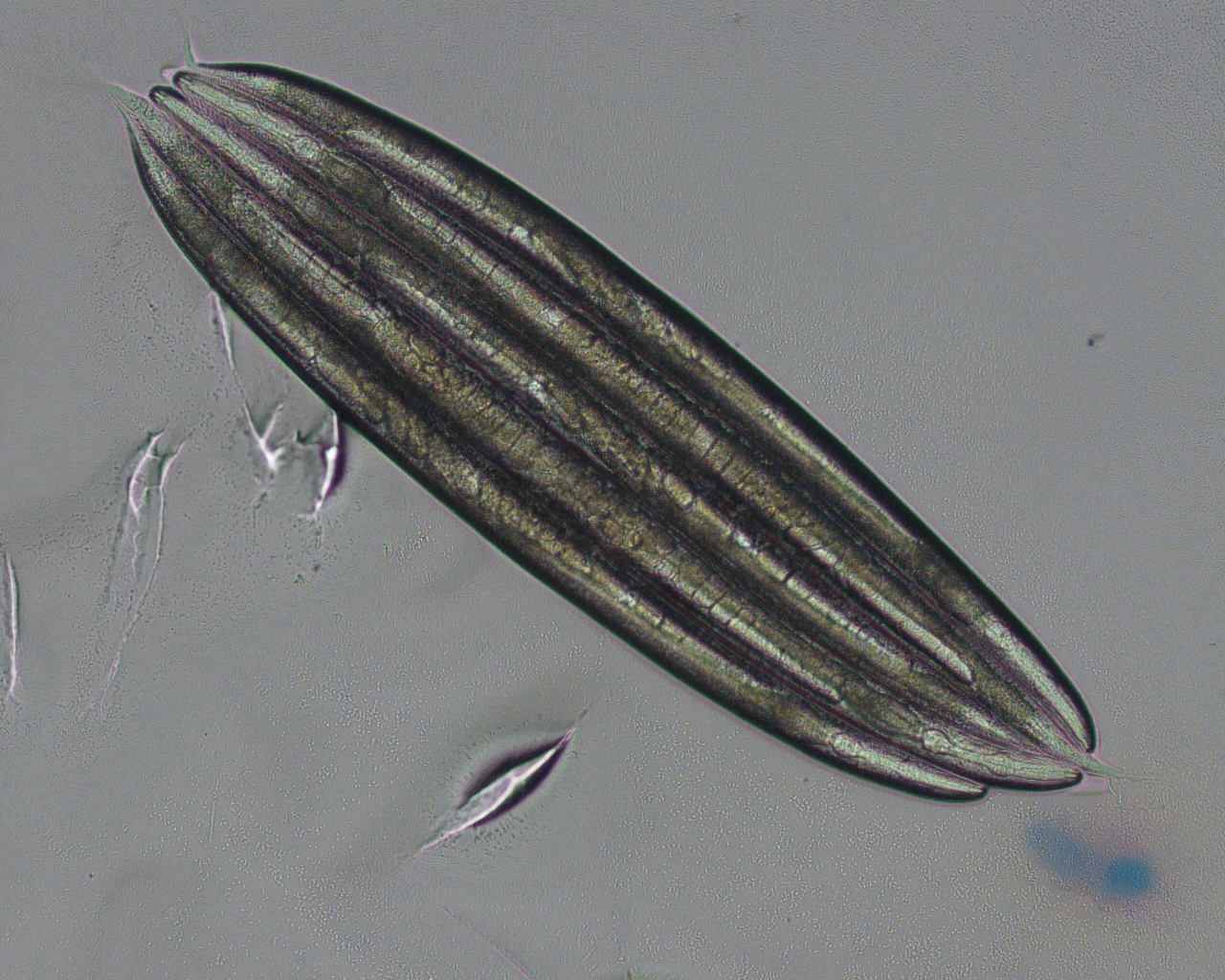

Supplement: S1 File — (ZIP) [file pone.0326100.s002.zip › Raw data of each experiment/Raw data Figure 2- Metformin effect on UPR/tmem-131/tmem-131 + MFM (BF).tif]

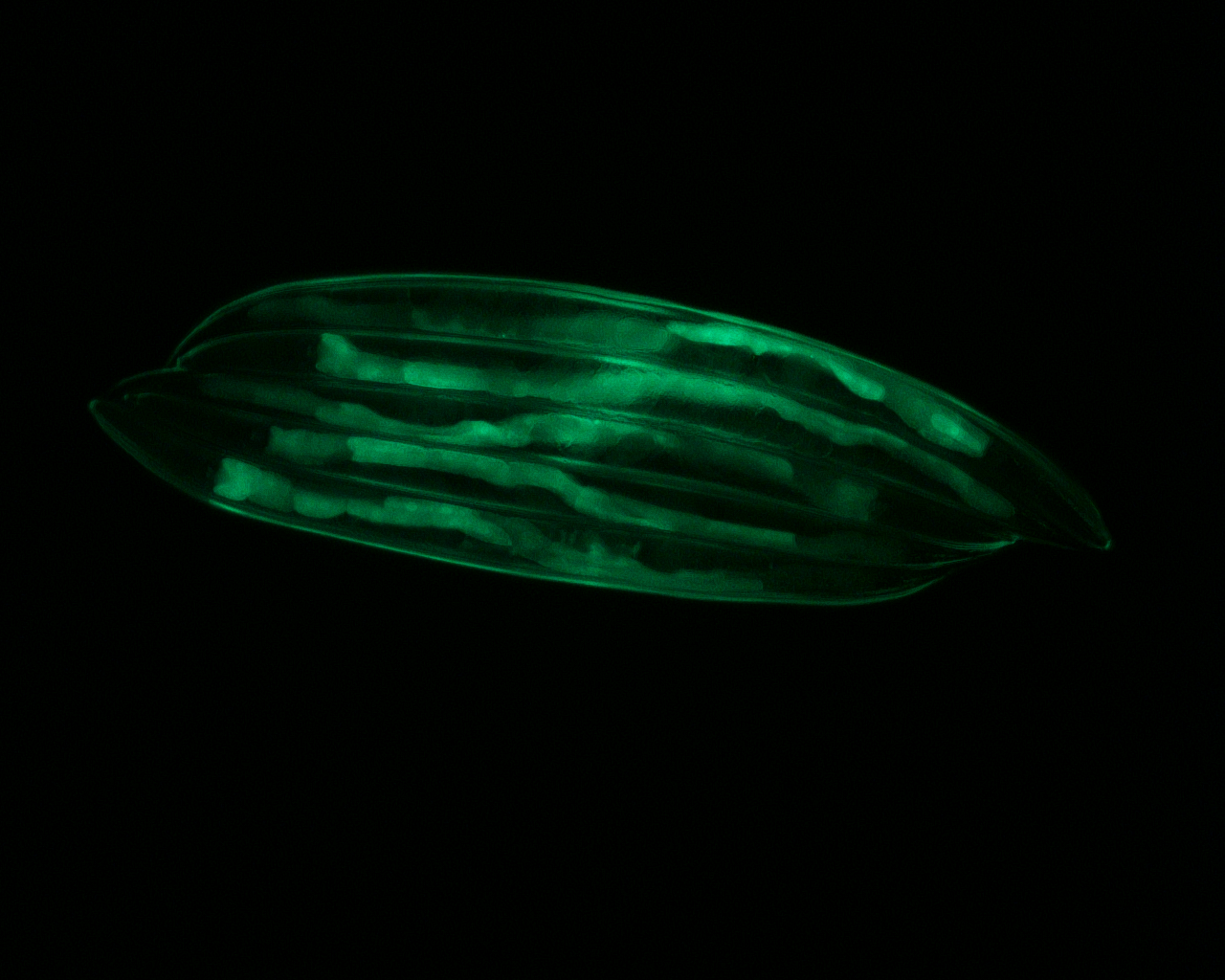

Supplement: S1 File — (ZIP) [file pone.0326100.s002.zip › Raw data of each experiment/Raw data Figure 2- Metformin effect on UPR/tomm-22/tomm-22 (GFP).tif]

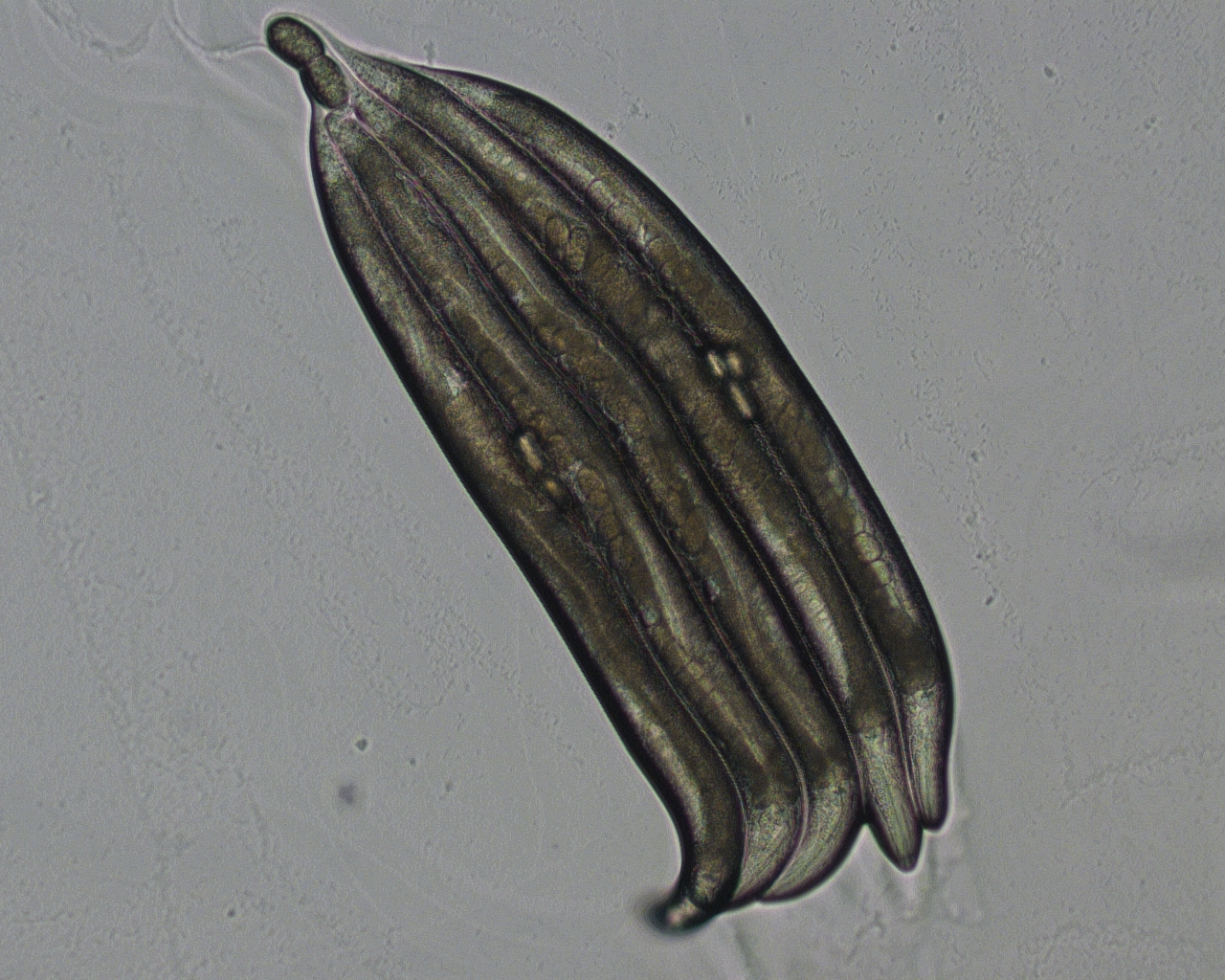

Supplement: S1 File — (ZIP) [file pone.0326100.s002.zip › Raw data of each experiment/Raw data Figure 2- Metformin effect on UPR/tomm-22/tomm-22 with MFM (BF).tif]

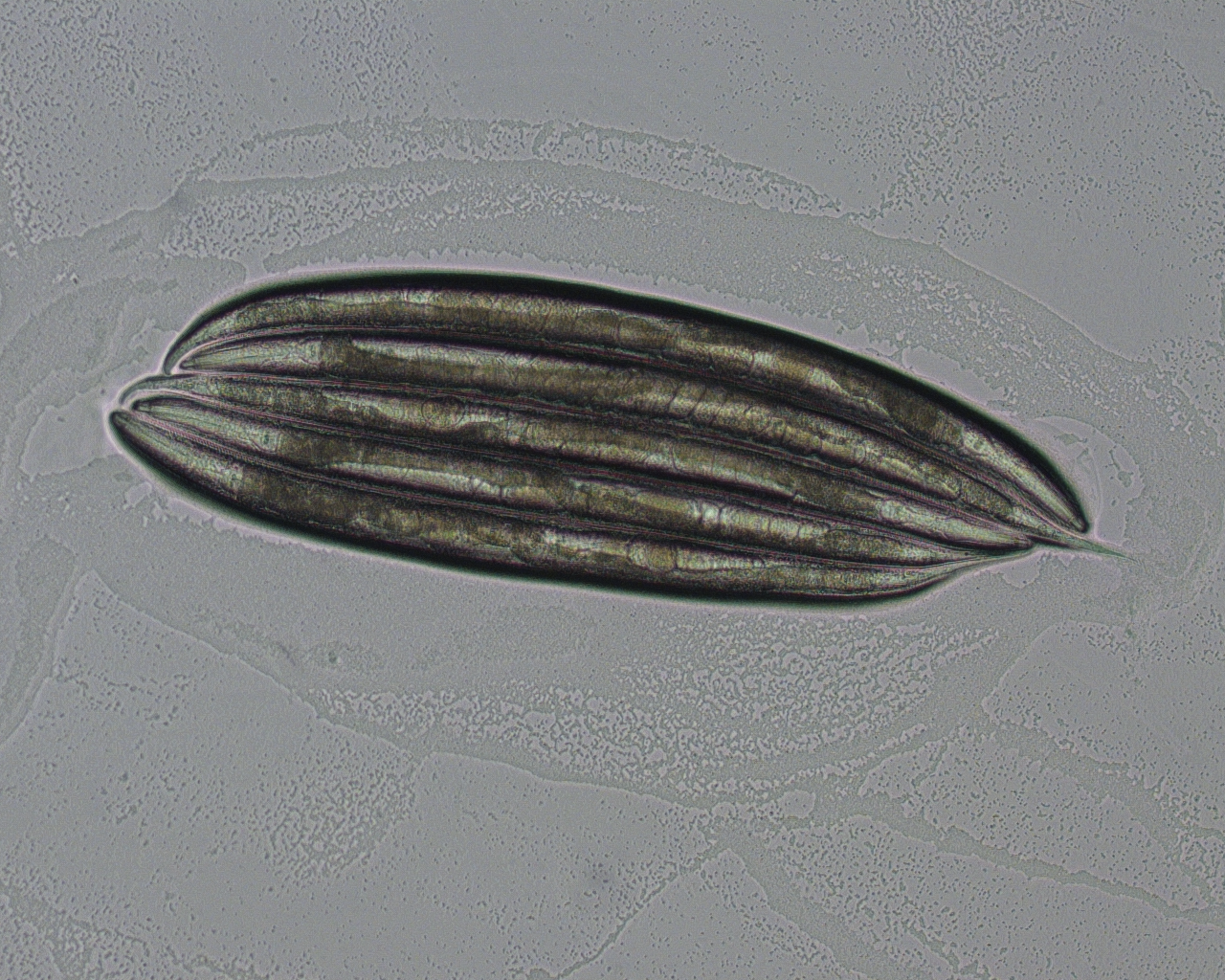

Supplement: S1 File — (ZIP) [file pone.0326100.s002.zip › Raw data of each experiment/Raw data Figure 2- Metformin effect on UPR/tomm-22/tomm-22 (BF).tif]

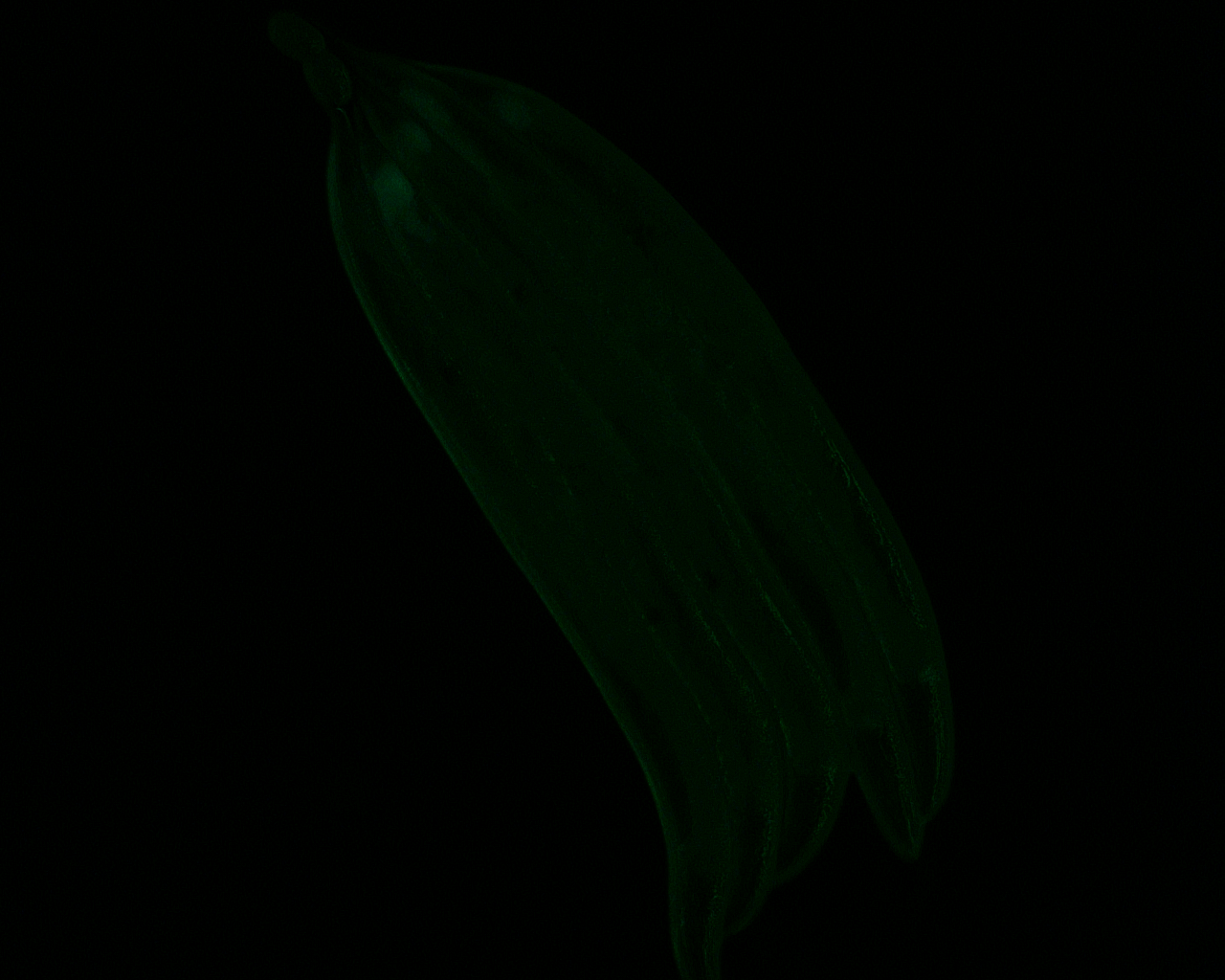

Supplement: S1 File — (ZIP) [file pone.0326100.s002.zip › Raw data of each experiment/Raw data Figure 2- Metformin effect on UPR/tomm-22/tomm-22 with MFM (GFP).tif]

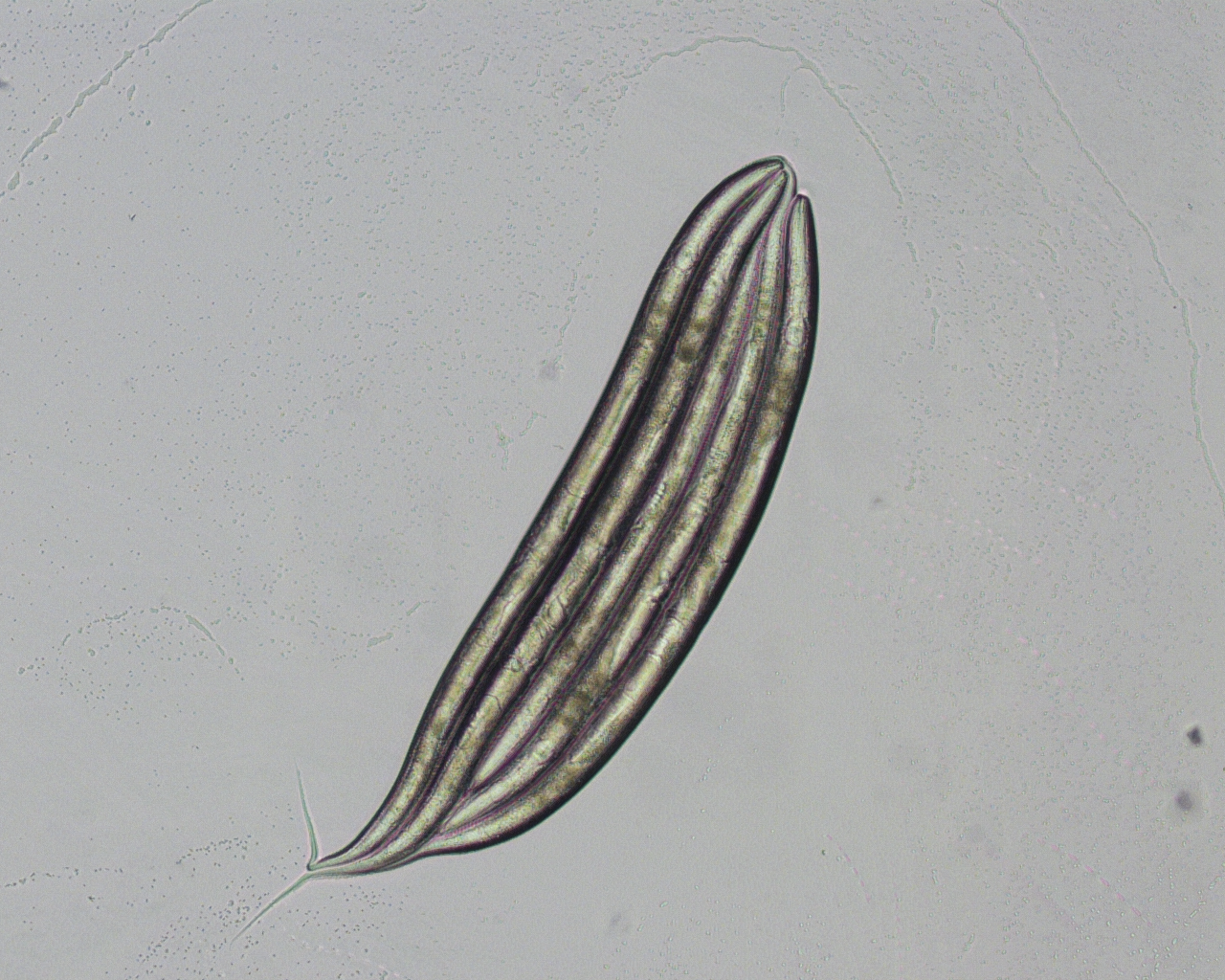

Supplement: S1 File — (ZIP) [file pone.0326100.s002.zip › Raw data of each experiment/Raw data Figure 2- Metformin effect on UPR/cco-1/cco-1 (BF).tif]

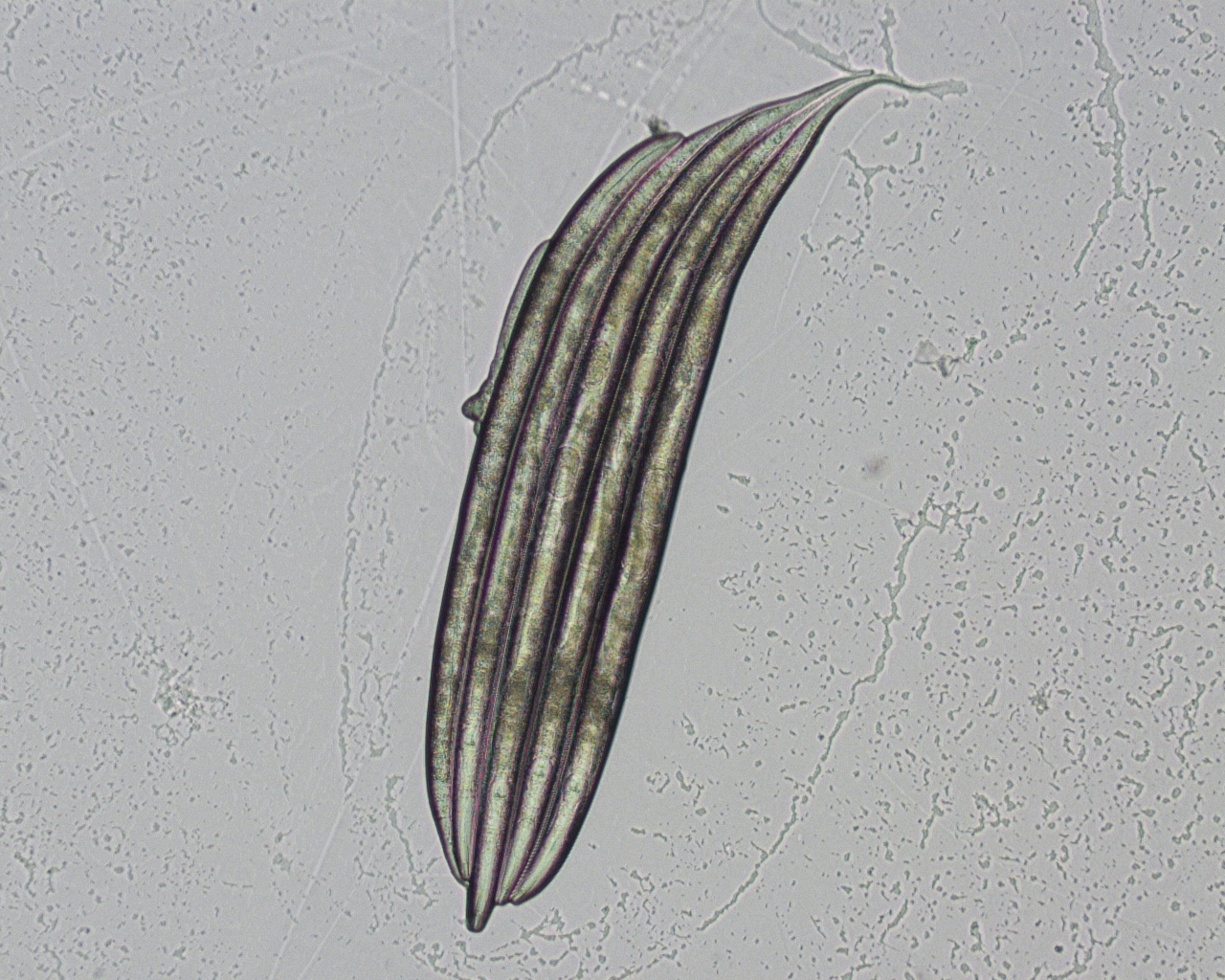

Supplement: S1 File — (ZIP) [file pone.0326100.s002.zip › Raw data of each experiment/Raw data Figure 2- Metformin effect on UPR/cco-1/cco-1 with MFM (BF).tif]

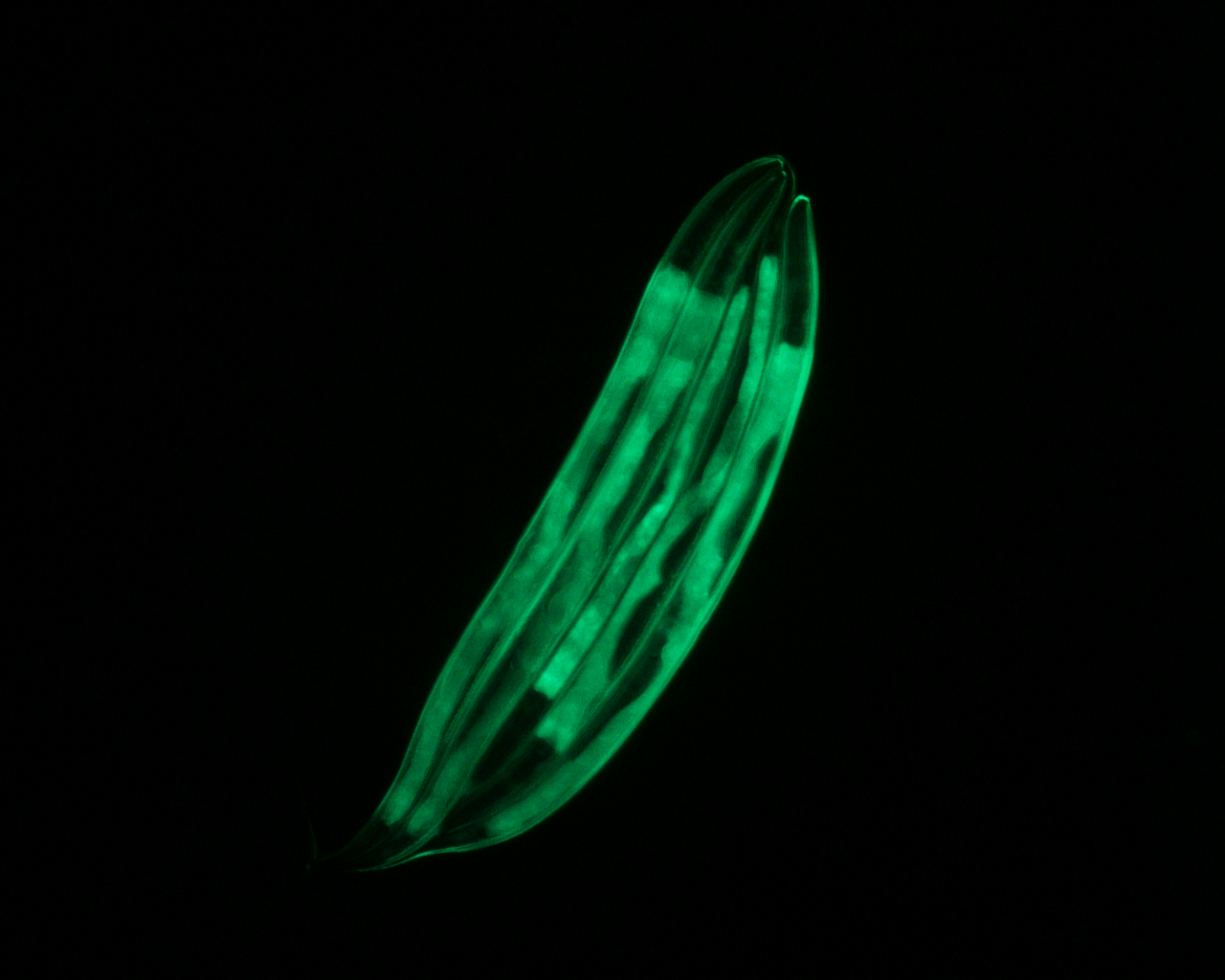

Supplement: S1 File — (ZIP) [file pone.0326100.s002.zip › Raw data of each experiment/Raw data Figure 2- Metformin effect on UPR/cco-1/cco-1 (GFP).tif]

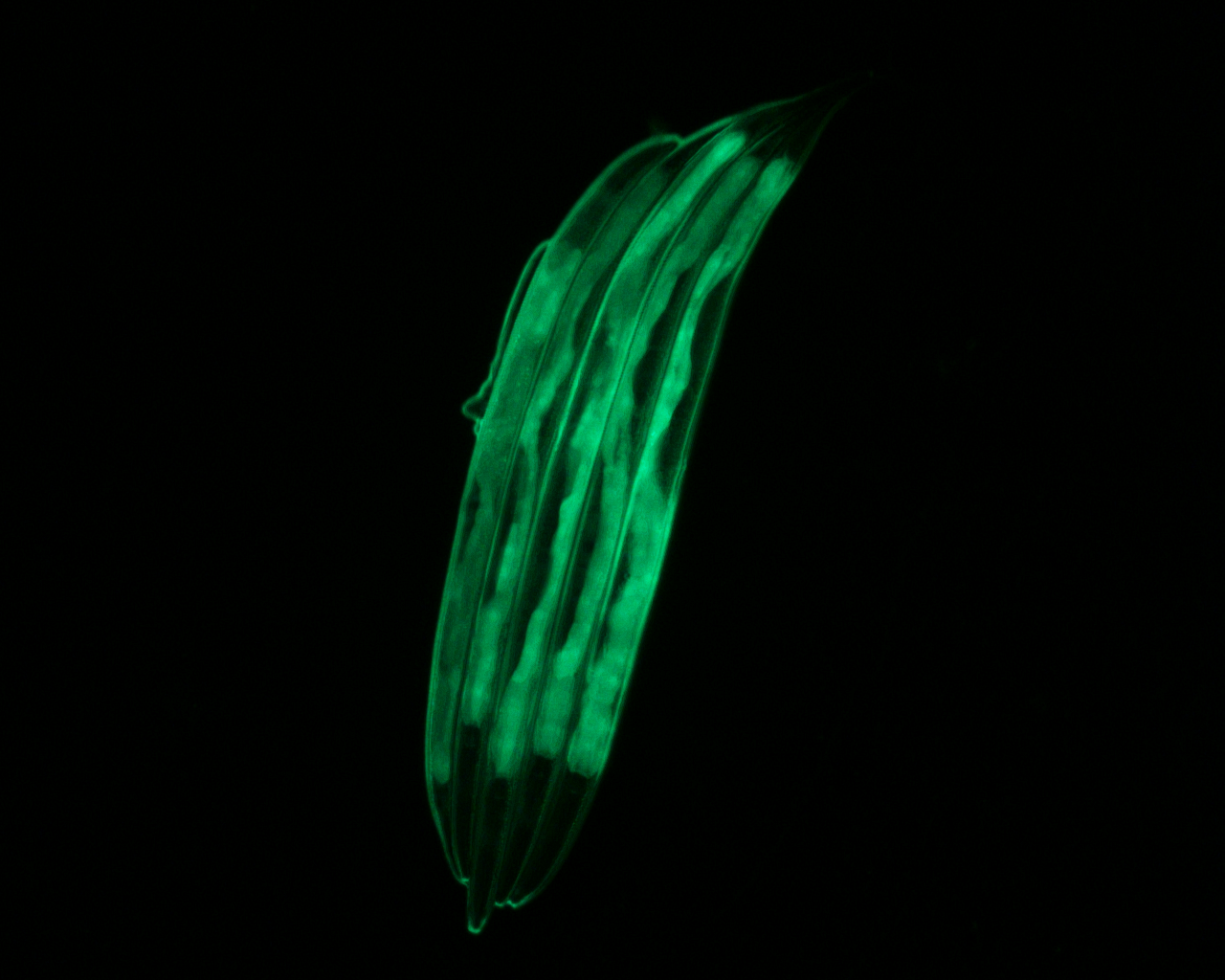

Supplement: S1 File — (ZIP) [file pone.0326100.s002.zip › Raw data of each experiment/Raw data Figure 2- Metformin effect on UPR/cco-1/cco-1 with MFM (GFP).tif]

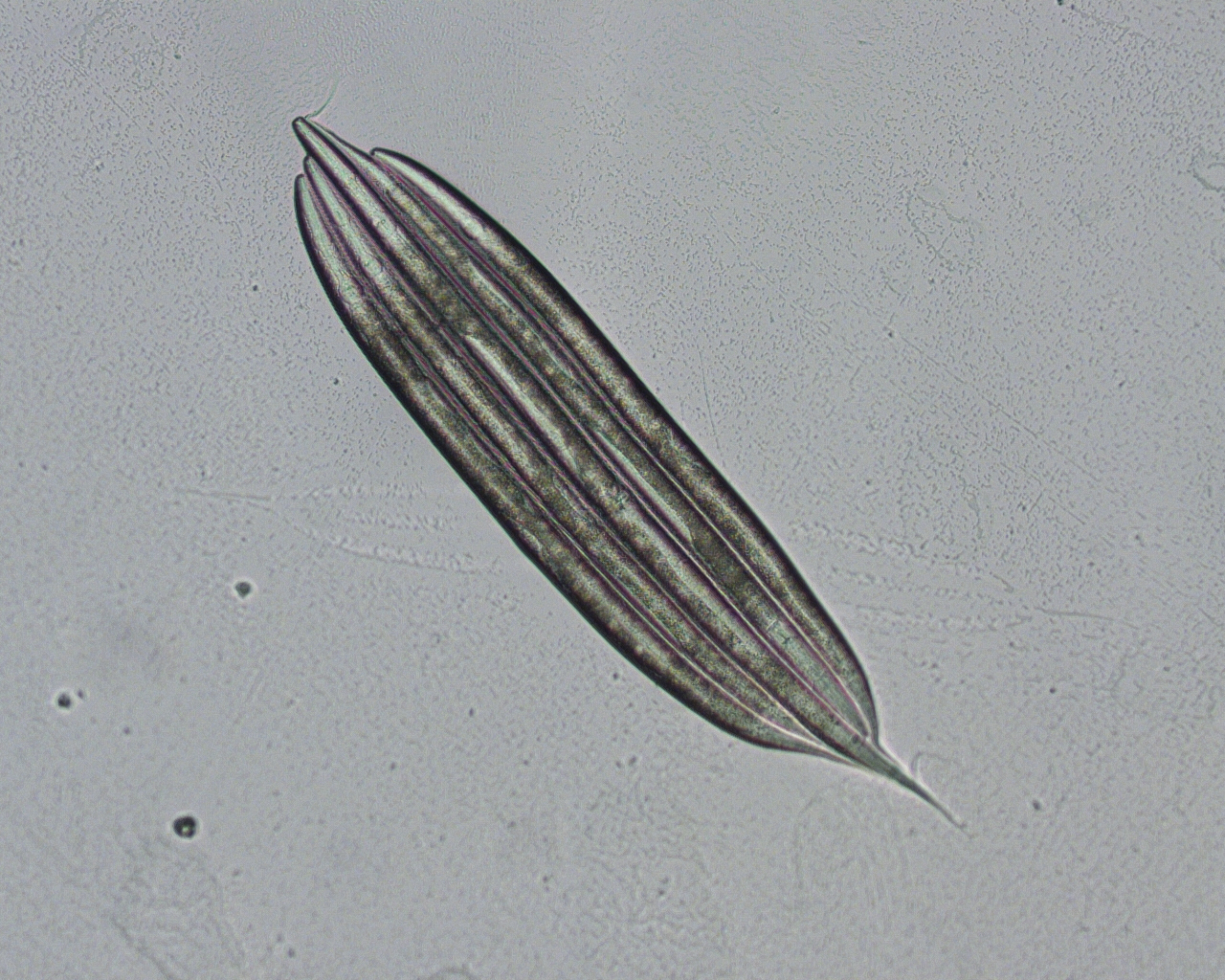

Supplement: S1 File — (ZIP) [file pone.0326100.s002.zip › Raw data of each experiment/Raw data Figure 2- Metformin effect on UPR/nuo-6/nuo-6 with MFM (BF).jpg]

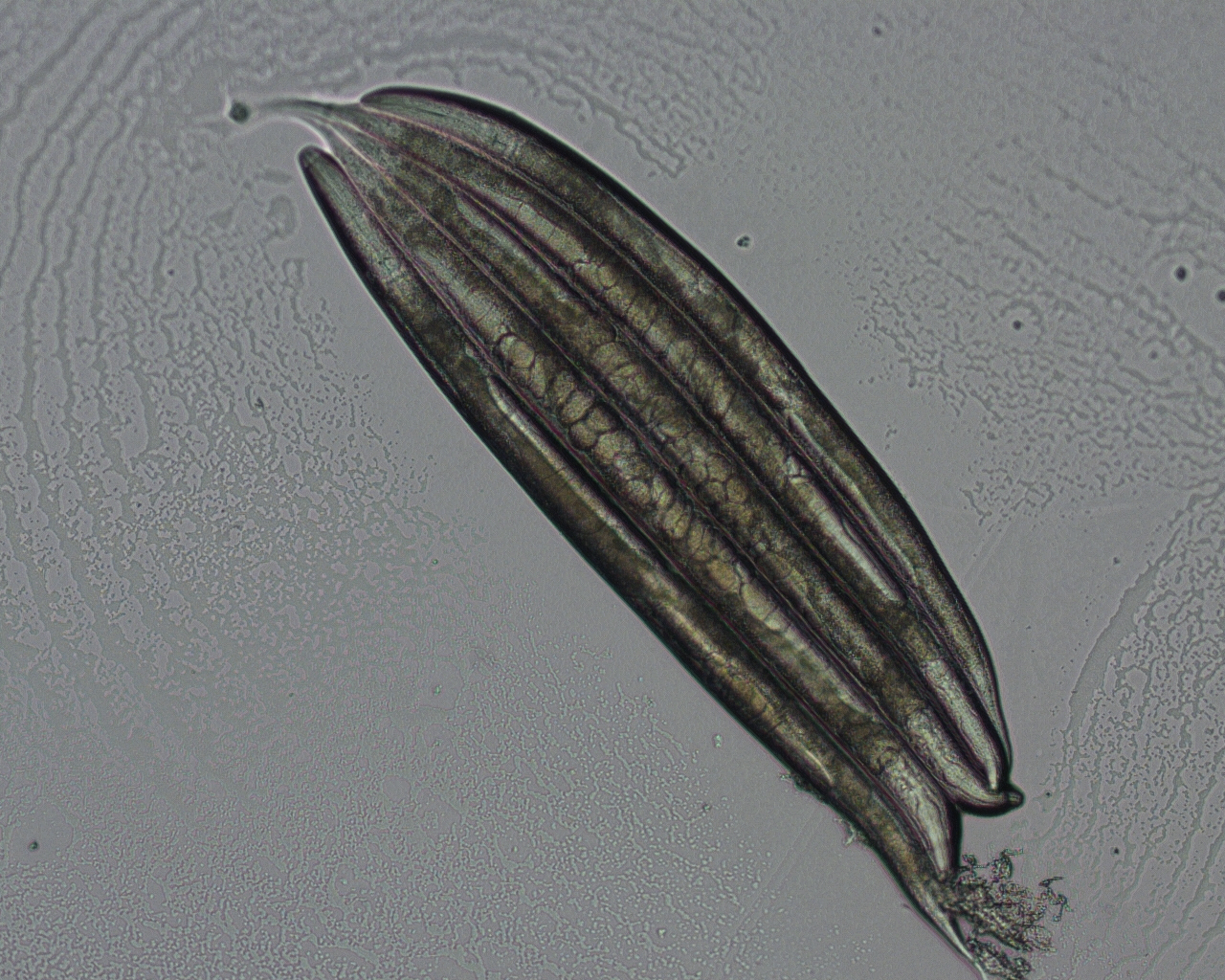

Supplement: S1 File — (ZIP) [file pone.0326100.s002.zip › Raw data of each experiment/Raw data Figure 2- Metformin effect on UPR/nuo-6/nuo-6 (BF).jpg]

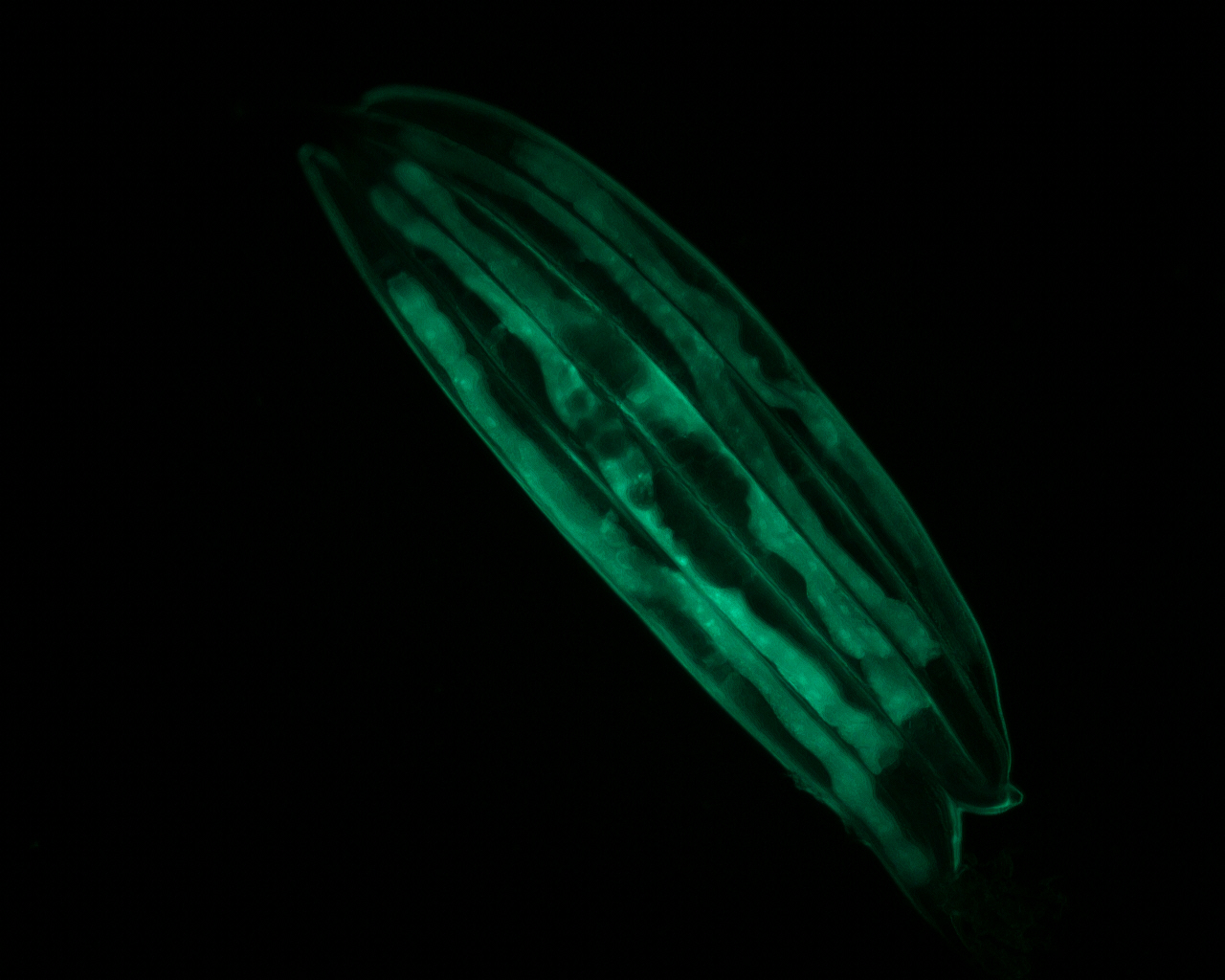

Supplement: S1 File — (ZIP) [file pone.0326100.s002.zip › Raw data of each experiment/Raw data Figure 2- Metformin effect on UPR/nuo-6/nuo-6 (GFP).jpg]

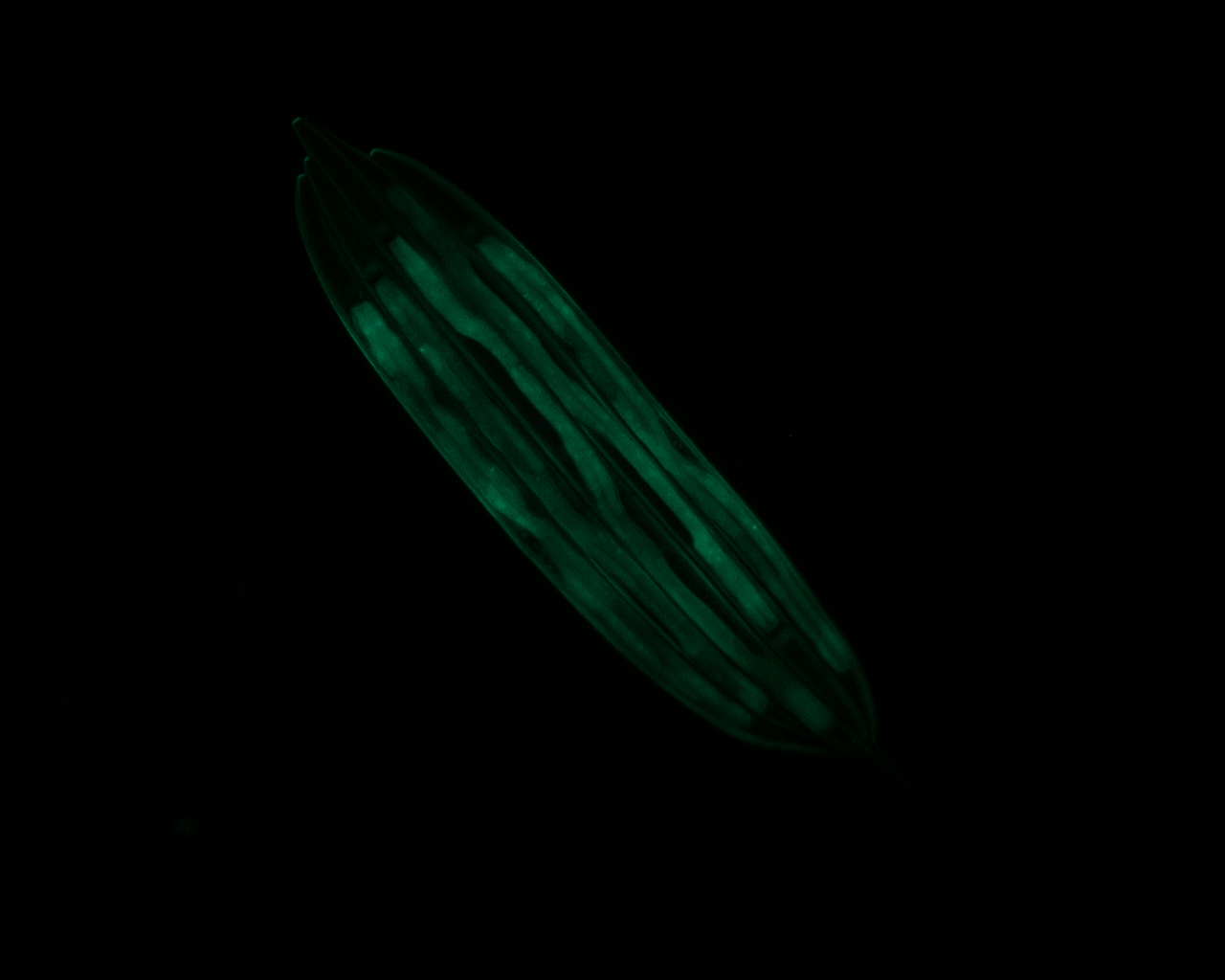

Supplement: S1 File — (ZIP) [file pone.0326100.s002.zip › Raw data of each experiment/Raw data Figure 2- Metformin effect on UPR/nuo-6/nuo-6 with MFM (GFP).jpg]

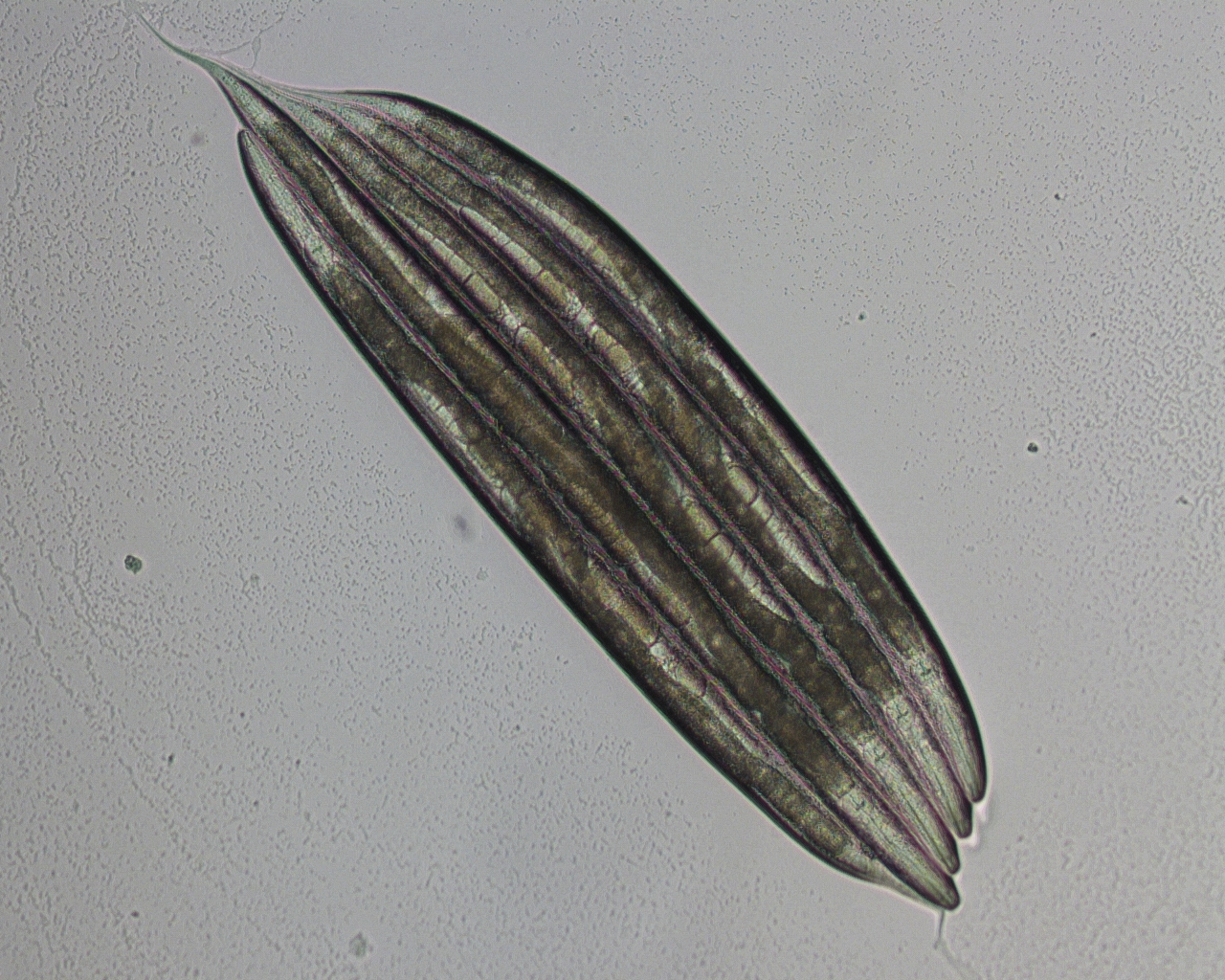

Supplement: S1 File — (ZIP) [file pone.0326100.s002.zip › Raw data of each experiment/Raw data Figure 2- Metformin effect on UPR/hsp-6 GFP (UPRmt)/hsp-6 (BF).tif]

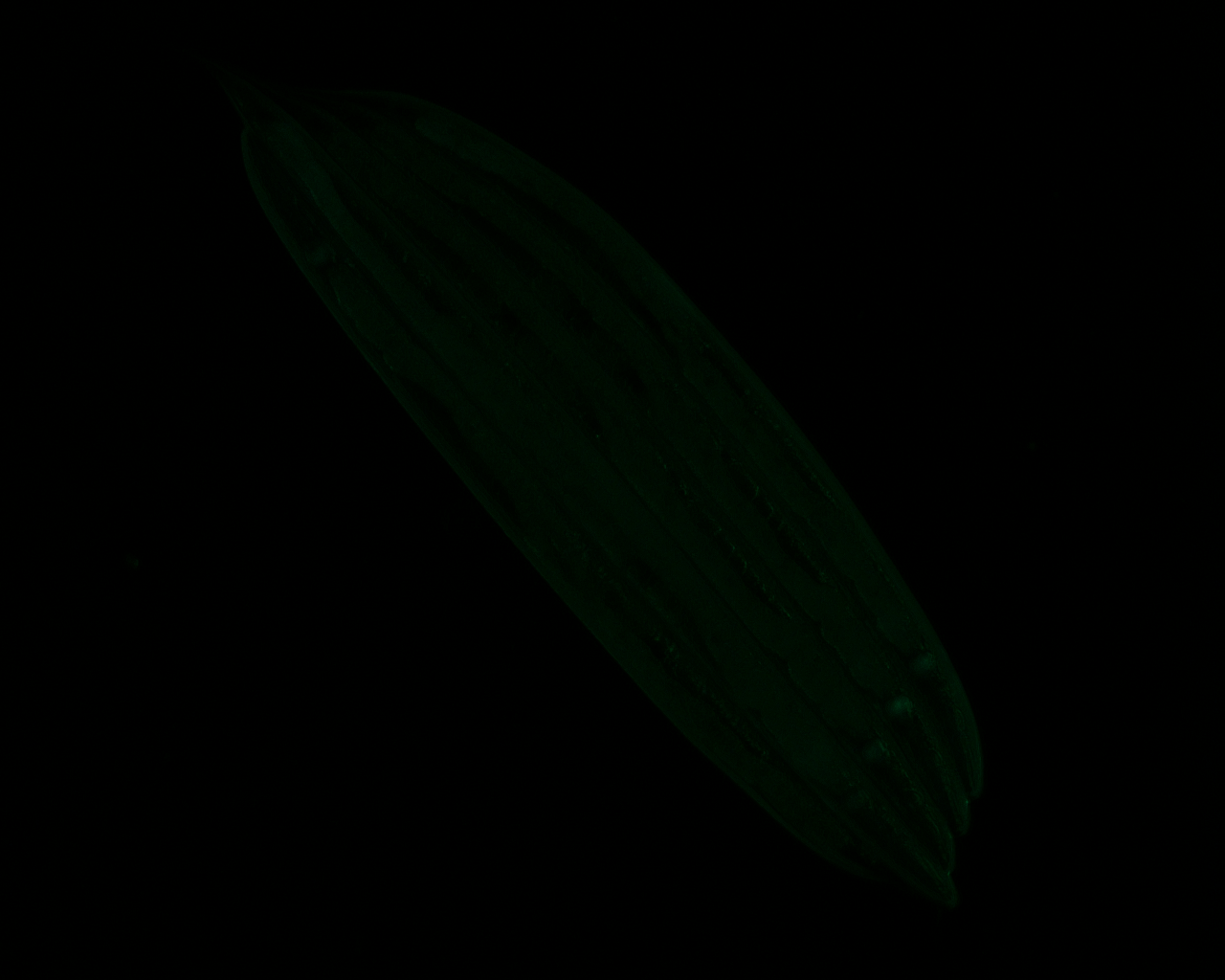

Supplement: S1 File — (ZIP) [file pone.0326100.s002.zip › Raw data of each experiment/Raw data Figure 2- Metformin effect on UPR/hsp-6 GFP (UPRmt)/hsp-6 (GFP).tif]
