# Supplementary material for: Metformin modulates the unfolded protein responses, altering lifespan and health-promoting effects in UPR-activated worms
Source: PLoS One. 2025 Jun 16;20(6):e0326100. doi: 10.1371/journal.pone.0326100 (PMC12169583; doi:10.1371/journal.pone.0326100)
Supplement: Attachment — Submitted filename: Rebuttal Letter.pdf [file pone.0326100.s003.pdf]

Rebuttal Letter

Dear Editor,

We express our sincere gratitude for the decision to revise our manuscript titled "Metformin Modulates the Mitochondrial Unfolded Protein Response, Altering Lifespan and Health-Promoting Effects in UPR-Activated Worms", decision: revision required PONE-D-25-06689 - [EMID:6c45c24e68a8ebbd]. We have carefully reviewed the editor's and reviewers' feedback and have made a comprehensive effort to address their concerns as outlined.

Journal Requirements:

When submitting your revision, we need you to address these additional requirements.

1. Please ensure that your manuscript meets PLOS ONE's style requirements, including those for file naming. The PLOS ONE style templates can be found at https://journals.plos.org/plosone/s/file?id=wjVg/PLOSOne_formatting_sample_main_body.pdf and https://journals.plos.org/plosone/s/file?id=ba62/PLOSOne_formatting_sample_title_authors_affiliations.pdf

Response: We meticulously ensure that the revised manuscript conforms to the style guidelines prescribed by PLOS ONE.

2. We note that the grant information you provided in the 'Funding Information' and 'Financial Disclosure' sections do not match.

When you resubmit, please ensure that you provide the correct grant numbers for the awards you received for your study in the 'Funding Information' section.

Response: The previously provided information has been precisely updated upon our resubmission.

3. Thank you for stating the following in the Acknowledgments Section of your manuscript:
This work is supported by National Research Council of Thailand (NRCT) and Mahidol University (NRCT5-RSA63015-20). Some strains were provided by the CGC, which is funded by NIH Office of Research Infrastructure Programs (P40 OD010440). We would like to thank Brian Onken from Rutgers, The State University of New Jersey, and Profs. Phil Morgan and Margaret Sedensky from University of Washington for their valuable inputs.

We note that you have provided funding information that is not currently declared in your Funding Statement. However, funding information should not appear in the Acknowledgments section or other areas of your manuscript. We will only publish funding information present in the Funding Statement section of the online submission form. Please remove any funding-related text from the manuscript and let us know how you would like to update your Funding Statement. Currently, your Funding Statement reads as follows:
- Author who received the fund: WS

- Funder and grant number: National Research Council of Thailand (NRCT) and Mahidol University (NRCT5-RSA63015-20)

- URL of funder website: https://www.nrct.go.th/

- C. elegans strains were provided by the CGC, which is funded by NIH Office of Research Infrastructure Programs (P40 OD010440).

** The funders absolutely did not play any role in the study design, data collection and analysis, decision to publish, or preparation of the manuscript**

Please include your amended statements within your cover letter; we will change the online submission form on your behalf.

Response: We apologize for any inconvenience caused. The funding statement provided in the initial submission contained accurate information regarding all funders. In addition, we have decided to omit the “Acknowledge” section from the revised manuscript.

4. We note that your Data Availability Statement is currently as follows: All relevant data are within the manuscript and its Supporting Information files.

Please confirm at this time whether or not your submission contains all raw data required to replicate the results of your study. Authors must share the “minimal data set” for their submission. PLOS defines the minimal data set to consist of the data required to replicate all study findings reported in the article, as well as related metadata and methods (https://journals.plos.org/plosone/s/data-availability#loc-minimal-data-set-definition).

For example, authors should submit the following data:

- The values behind the means, standard deviations and other measures reported;
- The values used to build graphs;
- The points extracted from images for analysis.

Authors do not need to submit their entire data set if only a portion of the data was used in the reported study.

If your submission does not contain these data, please either upload them as Supporting Information files or deposit them to a stable, public repository and provide us with the relevant URLs, DOIs, or accession numbers. For a list of recommended repositories, please see https://journals.plos.org/plosone/s/recommended-repositories.

If there are ethical or legal restrictions on sharing a de-identified data set, please explain them in detail (e.g., data contain potentially sensitive information, data are owned by a third-party organization, etc.) and who has imposed them (e.g., an ethics committee). Please also provide contact information for a data access committee, ethics committee, or other institutional body to which data requests may be sent. If data are owned by a third party, please indicate how others may request data access.

Response: The requisite dataset has been uploaded as Supporting Information files in our resubmission.

5. We note you have included a table to which you do not refer in the text of your manuscript. Please ensure that you refer to Table 1 in your text; if accepted, production will need this reference to link the reader to the Table.

Response: Table 1 has been referenced in the revised manuscript’s text.

Reviewers' comments:

Reviewer's Responses to Questions

Comments to the Author

1. Is the manuscript technically sound, and do the data support the conclusions?

The manuscript must describe a technically sound piece of scientific research with data that supports the conclusions. Experiments must have been conducted rigorously, with appropriate controls, replication, and sample sizes. The conclusions must be drawn appropriately based on the data presented.

Reviewer #1: Partly

---

2. Has the statistical analysis been performed appropriately and rigorously?

Reviewer #1: Yes

---

3. Have the authors made all data underlying the findings in their manuscript fully available?

The PLOS Data policy requires authors to make all data underlying the findings described in their manuscript fully available without restriction, with rare exception (please refer to the Data Availability Statement in the manuscript PDF file). The data should be provided as part of the manuscript or its supporting information, or deposited to a public repository. For example, in addition to summary statistics, the data points behind means, medians and variance measures should be available. If there are restrictions on publicly sharing data—e.g. participant privacy or use of data from a third party—those must be specified.

Reviewer #1: Yes

4. Is the manuscript presented in an intelligible fashion and written in standard English?

PLOS ONE does not copyedit accepted manuscripts, so the language in submitted articles must be clear, correct, and unambiguous. Any typographical or grammatical errors should be corrected at revision, so please note any specific errors here.

Reviewer #1: No

---

5. Review Comments to the Author

Please use the space provided to explain your answers to the questions above. You may also include additional comments for the author, including concerns about dual publication, research ethics, or publication ethics. (Please upload your review as an attachment if it exceeds 20,000 characters)

Reviewer #1: The manuscript by Tan and colleagues explores the effects of metformin on lifespan, stress resistance, and locomotion in C. elegans and its relationship with Unfolded Protein Response (UPRmt and UPRer) pathways. In particular, the study demonstrates differential effects on UPRmt in tomm-22 and cco-1 RNAi worms and presents a complex interaction between metformin treatment and stress responses. While the topic is timely and the study potentially offers new insights into the metformin’s action on C. elegans longevity, several aspects of the manuscript require clarification and/or further experimental support.

Major Comments:

1. The manuscript states that "metformin's action is mitochondria-specific" and that "UPRmt is indispensable for lifespan extension." However, the data (especially the lifespan extension observed in both UPRmt- and UPRer-activated strains) suggest that these statements may be overgeneralized. Only two representative RNAi inducer per UPR pathway (tomm-22 and cco-1 for UPRmt; mdt-15 and tmem-131 for UPRer) has been tested. It would strengthen the study to include additional examples of UPRmt and UPRer inducers to verify whether the observed patterns are robust.

Response: We acknowledge your concern regarding this matter. After careful deliberation and the acquisition of additional experiments (in new Figure 2), we have modified our interpretation in the revised manuscript. To enhance the comprehensiveness of our analysis regarding the interaction between metformin and the UPRs, we also tested additional UPR-activated worms. These included *nuo-6* RNAi (UPR^{mt}) and *tag-335* RNAi (UPR^{er}) worms, whose lifespan and stress resistance phenotypes, however, were not carried on as in others. Our new data indicates that metformin exhibits the ability to affect both UPR^{mt}- and UPR^{er}-activated strains. This is discussed in the Discussion section of the revised manuscript.

2. The authors should clarify whether they mean that the beneficial effect in tomm-22 RNAi-treated worms is due to metformin resolving mitochondrial stress or if alternative mechanisms might be at play. What do authors believe it is the mechanism behind metformin action, is a decrease on protein translation that is alleviating the stress on tomm-22 RNAi-treated worms?

Response: We demonstrated the beneficial effects of metformin in *tomm-22* RNAi-treated worms, including lifespan extension (Fig. 1C) and promoting late-life oxidative stress resistance (Fig 4B and Table 3).

In the context of lifespan alteration, the activation of UPR^{mt} in *tomm-22* worms resulted in a shortened lifespan (Fig. 1C). However, metformin demonstrated the ability to enhance the lifespan of these worms. To elucidate the underlying mechanism, We conducted a new experiment (presented in Supplementary) in which we inactivated the transcription factor and regulator of the UPR^{mt}, ATFS-1 and UBL-5, in metformin-fed *tomm-22* worms using RNAi knockdown. This experiment aimed to validate our hypothesis that the lifespan extension observed with metformin in *tomm-22* worms is UPR^{mt}-independent. The results indicated that when UPR^{mt} is inactivated, the worms exhibited a significant lifespan extension compared to those fed metformin. This suggests that the activation of UPR^{mt} in *tomm-22* has a detrimental impact (possibly due to its other secondary effects) on the worm's lifespan, which metformin can counteract.

Activation UPR^{mt} in *tomm-22* can theoretically activate the proteomic stress response, which should benefit the worm's antioxidant system in mitochondria. However, the direct effect of *tomm-22* knockdown is the impairment of the mitochondrial transport system, as previously addressed in the Discussion. Consequently, mitochondrial protein chaperones may not be able to function at their full capacity. Notably, metformin was shown to promote late-life oxidative stress resistance (Fig. 4B and Table 3). Therefore, metformin may exhibit additional unknown effects that mitigate the impairment of the mitochondrial transport system, facilitating the entry of protein chaperones into mitochondria and ultimately enhancing the antioxidant system.

3. The discussion suggests that metformin's beneficial effects on lifespan are independent of UPRmt/UPRer activation. This conclusion partially conflicts with previous work ( ), which show that ubl-5 is required for lifespan induced by metformin in C. elegans. A discussion on how the present findings compare and contrast with this previous paper is necessary. Alternatively, authors could do the assay with RNAi against ubl-5. This would be important to support their claim that metformin action on lifespan is not dependent on UPRmt

Response: The previous work by Espada *et al.*, 2020, investigated the role of *ubl-5*, *atfs-1* and *isp-1* in metformin mediated longevity and primarily focused on mitochondrial stress signaling and UPR^{mt}. Their results on *atfs-1* and *isp-1* knockdown shortened lifespan of the nematodes but more importantly when treated with metformin, shortened lifespan further and exacerbated metformin toxicity. *ubl-5*, however, did not have a significant effect and thus is reported to be required for lifespan extension.

It is important to note that a previous study by Benedetti *et al*., 2006, Figure 3A, showed that *ubl-5* knockdown did not induce *hsp-6* and *hsp-60*::GFP in heat stress, suggesting that *ubl-5* is required for the initiation of UPR^{mt} and subsequent activation of chaperones. In contrast, the primary role of *tomm-22* is a mitochondrial outer membrane transporter rather than a UPR^{mt} stress regulator. The activation of *hsp-6* response in our study was likely a response to changes in the mitochondrial import machinery instead of a direct regulatory response to mitochondrial stress.

As suggested by the reviewer, we did another experiment to investigate whether *tomm-22* and *ubl-5* have overlapping pathways by performing RNAi knockdown on both genes and treating the worms with metformin (Supplementary data, Fig. 1). Logically, if *ubl-5* was required for metformin-mediated longevity, then the effect of metformin would be lost in the presence of a *tomm-22* background.

We found that *tomm-22* and *ubl-5* double knockdown with metformin synergized and extended lifespan significantly when compared to *tomm-22* treatment control. These findings suggest that both *tomm-22* and *ubl-5* do not overlap in their pathways but also contest the notion that *ubl-5* is required for metformin-mediated longevity.

We argue that *ubl-5* is not required for metformin-mediated longevity and its mitochondrial impairment effects merely exacerbate toxicity of metformin instead, similar to *atfs-1* and *isp-1*. However, the disruption in mitochondrial import machinery ameliorated these effects (likely by disrupting the flux of chaperone proteins and mitochondrial activity) and prevented mitochondrial impairment (as described by Espada), maintaining the metformin-mediated longevity effect.
It was also important to note that Espada *et al.* also mentioned that they tested whether UPR^{mt} was affected by metformin using *hsp-6*::GFP, and found that metformin administration did not lead to elevated expression of GFP nor reduction of mitochondrial protein levels. They have also cited De Haes *et al*., 2014, where the lack of UPR^{mt} induction by metformin was reported. In this case, our findings were in line with both Espada and De Haes, as we did find that there was no UPR^{mt} induction by metformin.

Essentially, our work highlights that metformin can extend lifespan without a strict dependency on UPR^{mt}, as we found that *cco-1* knockdown (impairing cytochrome c oxidase subunit 1) upregulated *hsp-6*::GFP response while also extending lifespan. The contrasting result from *tomm-22* further highlights the complexity of the relationship between mitochondrial stress and lifespan extension and emphasizes that UPR^{mt} is not an absolute requirement for metformin-mediated lifespan extension. Whether metformin-mediated lifespan extension being dependent on *ubl-5* is beyond the scope of this study.

4. Cabreiro et al., 2013 (PMID: 23540700) also reported that metformin extends C. elegans lifespan on OP50, but not on HT115. Could authors also please provide an explanation of the apparent discrepancy between this study and Cabreiro's work?

Response: It is indeed quite difficult to pinpoint the exact reason for the observed difference in lifespan results between our study and theirs, particularly because both studies were conducted under separate laboratory conditions which introduces variables that could influence the outcomes.

One possible factor that could explain the difference in lifespan would likely be the use of FUdR (5-fluoro-2'-deoxyuridine) in their experiments. In contrast to their approach, we omitted FUdR from our lifespan experiments to avoid potential confounding effects, as the impact of FUdR on lifespan has been shown to be significant.

The use of FUdR has been reported to affect gene expression patterns (McIntyre *et al.,* 2021) and metabolic profiles of *C. elegans* (Davies *et al*., 2012). FUdR works by inhibiting DNA synthesis, which leads to reduced cell proliferation particularly in the reproductive tissues, and in some cases has been associated with lifespan extension. The effect of FUdR on longevity is, however, context-dependent, and can vary due to the age and specific mutation background of the worms (Hosono *et al*., 1982, Aitlhadj and Sturzenbaum, 2010, Van Raamsdonk and Hekimi, 2011). Whether this effect affects OP50 or HT115 differentially remains to be determined, as this has not been studied yet.

We would like to highlight that various other experimental conditions could contribute to the differences in lifespan, including environmental factors like temperature, worm strain variations and differences in handling protocols, which may all impact the outcome of lifespan assays.

5. Figure 2C and 2E: The representative images of GFP fluorescence for the empty vector control appear inconsistent with the reported quantification. Moreover, a better description of how the fluorescence was analyzed would improve the manuscript. For example, in 2E are the values displayed a quantification per worm or of a whole section? Was there a normalization of the background for each image?

Response: The quantitative data may appear to differ from the representative images due to a background issue. We, unfortunately did not collect the brightfield images of the corresponding strains and conditions for background correction and worm contour correction.

However, the purpose of this experiment is to demonstrate the effect of metformin on UPR^{mt} and UPR^{er}. We conducted another experiment to address this purpose and present it as a new Figure 2 in the revision. We employed all UPR^{mt}- and UPR^{er}-activated strains acquired in our laboratory to assess the impact of metformin on adult day 1. Due to the transient nature of GFP expression, which diminishes over time as animals age, we refrained from monitoring its expression after a 3-day interval, as previously done. All images were captured using consistent camera settings to facilitate comparative analysis of GFP expression. The details of the GFP analysis have been meticulously documented in the Materials and Methods section of the revised manuscript.

6. Figure 5 shows that mdt-15 RNAi-treated worms exhibit increased movement when compared to N2 despite being short-lived. This is counterintuitive. Can authors please provide an explanation for this? The manuscript would also improve from a better methodological description of how crawling velocity was obtained.

Response: Our research objectives include investigating the impact of UPR activation on the lifespan and healthspan of worms. Our findings demonstrated that worms treated with *mdt-15* RNAi (UPR^{er}-activated) exhibited increased body bending, a measure of healthspan, on D6 (Fig 5A) despite a shortened lifespan (Fig 1E). In addition, we demonstrated that *tomm-22* RNAi (UPR^{mt}-activated) also exhibited increased body bending (Fig 5A) and increased crawling velocity (Fig 5E) despite a shortened lifespan (Fig 1C). These observations stand out as a significant finding in our study. This suggests that lifespan extension does not necessarily translate into improved health conditions, as evidenced by the healthspan metrics. Our study contributes to this observation, complementing the findings of Bansal *et al.*, 2015 (PMID: 25561524). This matter was also addressed in our revised manuscript.

Minor Comments:

1. The introduction section would benefit from a more detailed discussion of the molecular mechanisms involved in UPRmt and UPRer activation/regulation.

Response: Comprehensive details regarding the activation and regulation of UPR^{mt} and UPR^{er} have been incorporated into the Introduction section of the revised manuscript.

2. In ''These findings suggest that metformin's action is mitochondria-specific. Furthermore, they imply that UPRmt is indispensable for lifespan extension, as the metformin-induced reduction of UPRmt (Fig 2B) did not diminish its lifespan-extending effect''. I believe the authors meant to say "dispensable" instead of ''indispensable''. Please confirm the intended meaning, as the data suggest that even when UPRmt is suppressed (as in tomm-22 RNAi worms), lifespan extension still occurs.

Response: We sincerely appreciate your insightful comment. The appropriate term for the sentence is "dispensable". We have made the necessary correction in the revised manuscript.

3. The manuscript should clearly define what were the control conditions (N2/control) in all experiments. In some legend figures, it is not clear whether "control" refers to worms grown on OP50, HT115, or HT115 harboring L4440. Moreover, it was not clear what vehicle was used for metformin. Was it water? Was vehicle included on 0mM plates?

Response: We apologize for the confusion regarding the description of the control conditions that were pointed out by the reviewer. Clarifications have been incorporated in the revised manuscript and figure legends. In summary, the HT115 strain used in our experiment was the HT115 strain harboring the empty vector L4440, as indicated by the term "EV" in the revised manuscript. Most of the N2 control is the N2-Bristol wild-type strain grown on the EV. We used OP50 fed on N2 in lifespan assay only in Fig 1A.

In all experiments, sterile water was utilized as the vehicle for metformin administration.

4. The quantitative data in Figure 1, Figure 2 should include statistical information on the figure rather than relying solely on the information displayed on tables.

Response: The statistical significance of the data, as indicated by * has been labeled in the figures 1 and 2 as suggested. Tables 1 and 2 have also been modified accordingly.

5. There are some typos in the text and it should be double-checked for spelling (e.g., legend of figure 1 ''ifespan'' instead of ''lifespan'').

Response: The typographical errors have been carefully corrected in the revised manuscript.

6. PLOS authors have the option to publish the peer review history of their article (what does this mean?). If published, this will include your full peer review and any attached files.

If you choose "no", your identity will remain anonymous but your review may still be made public.

Response: We accept the provided option.

Do you want your identity to be public for this peer review? For information about this choice, including consent withdrawal, please see our Privacy Policy.

Reviewer #1: No

Our responses have concluded. The responses have been incorporated into the revised manuscript, ensuring their relevance and effectiveness. We value your time and effort in critiquing our work and assisting us in its refinement. We would be grateful if our resubmission and all provided responses met the publication requirements and satisfied all scientific considerations.

Thank you,

The authors
